# Traditional Chinese medicine in influenza treatment: a bibliometric analysis integrating multiple databases

**DOI:** 10.3389/fmicb.2026.1761339

**Published:** 2026-03-12

**Authors:** Fangkai He, Jing Xu, Tianlun Yu, Xingyu Zhu, Xiaolei Wang, Ji Yang, Yifei Xia, Fengmei Wu, Shicheng Su

**Affiliations:** 1 Department of Respiratory and Critical Care Medicine, Kunshan Hospital of Traditional Chinese Medicine, Kunshan, Jiangsu, China; 2 Department of Clinical Laboratory, Kunshan Rehabilitation Hospital, Kunshan, Jiangsu, China; 3 Department of Laboratory Medicine, The First Hospital of China Medical University, Shenyang, China

**Keywords:** bibliometrics, hotspot analysis, immune regulation, influenza, traditional Chinese medicine

## Abstract

**Background:**

Influenza, a highly contagious respiratory disease, is especially severe for the elderly, children, and immunocompromised individuals. Traditional Chinese medicine (TCM), with its antiviral, immune-modulating, and symptom-relieving properties, has gained attention as a potential treatment. This study uses bibliometric analysis to assess the research trends, hotspots, and progress of TCM in treating influenza.

**Methods:**

Literature from the Web of Science Core Collection (WOSCC), Scopus, and PubMed was analyzed using CiteSpace, VOSviewer, and Bibliometrix to explore author collaboration, research trends, clinical trials, and key advancements in TCM for influenza.

**Result:**

Research on TCM for influenza has steadily increased since 2000, with a marked surge post-2019 following the COVID-19 pandemic. China leads the field, contributing nearly two-thirds of the publications. Research focuses on TCM interventions, antiviral mechanisms, and immune modulation, with emerging hotspots in network pharmacology and molecular mechanisms.

**Conclusion:**

The study shows a steady annual growth rate of 16.94%, reflecting global interest in TCM for respiratory viral infections. Despite China’s leadership, international collaboration remains limited (10.23%). Research has shifted from empirical formulations to modern scientific methods, but further large-scale trials are needed to confirm TCM’s efficacy.

## 1 Introduction

Influenza, an acute respiratory infectious disease caused by the influenza virus, is primarily characterized by upper respiratory tract inflammation, excessive immune response activation, and viral-induced pulmonary damage (Herold et al., 2015). The influenza virus belongs to the Orthomyxoviridae family and is an RNA virus with a single-stranded, negative-sense genome composed of eight segments of genetic material. These segments include the polymerase basic proteins PB1 and PB2, the acidic polymerase PA, hemagglutinin (HA), nucleoprotein (NP), neuraminidase (NA), matrix protein (M), and nonstructural proteins (NS) (Webster et al., 1992). Based on the composition of NP and M, influenza viruses are classified into three types (A, B, and C) (Zheng et al., 2014). Influenza annually imposes a significant global health burden, particularly affecting the elderly, children, and immunocompromised individuals (GBD 2021 Lower Respiratory Infections and Antimicrobial Resistance Collaborators, 2024). According to the World Health Organization (WHO), influenza epidemics can result in millions of severe cases and hundreds of thousands of deaths (Hay and McCauley, 2018). Current treatment methods primarily rely on antiviral agents, such as neuraminidase inhibitors oseltamivir and zanamivir; however, their efficacy is limited due to issues of drug resistance and adverse effects, and their impact on alleviating influenza complications remains inconclusive (Javanian et al., 2021; Chan and Hui, 2023). As such, the development of novel therapeutic strategies remains a pressing challenge in the field of influenza prevention and treatment.

In recent years, traditional Chinese medicine (TCM) has emerged as a focal point in influenza treatment research, owing to its unique antiviral, immune-modulatory, and symptom-relieving properties (Mousa, 2017). TCM offers a new avenue for influenza treatment through multiple mechanisms, such as modulating immune responses, inhibiting viral replication, and alleviating inflammatory responses (Huang et al., 2021). Chinese herbal formulas, such as Lianhua Qingwen and Chaihu Shugan San, have demonstrated significant clinical efficacy, particularly in alleviating influenza symptoms and shortening the duration of illness (Zhan et al., 2025). However, the diverse forms of TCM applications, coupled with the lack of standardized efficacy evaluation criteria and insufficient verification of drug safety, have posed challenges to its clinical use. These limitations underscore the need for a systematic review of the progress and challenges in TCM-based influenza treatment.

Despite advancements in both clinical and fundamental research on TCM for influenza, bibliometric analyses in this field remain scarce. Existing studies often rely on a single database, overlooking the advantages of integrated database searches, resulting in incomplete data coverage and an inability to fully reflect the dynamic research trends. Furthermore, most bibliometric studies tend to focus on quantitative indicators, such as publication volume and author collaboration, with insufficient attention to in-depth analyses of clinical trial design, efficacy evaluation, and drug safety. To address these gaps, this study integrates data from multiple databases, including the Web of Science Core Collection (WOSCC), Scopus, and PubMed, and employs visualization tools to provide a comprehensive assessment of the research landscape on TCM for influenza. Additionally, this study aims to uncover key scientific issues and technological bottlenecks within the field, offering valuable insights for future research endeavors.

## 2 Methods

### 2.1 Data retrieval

**Figure 1:**
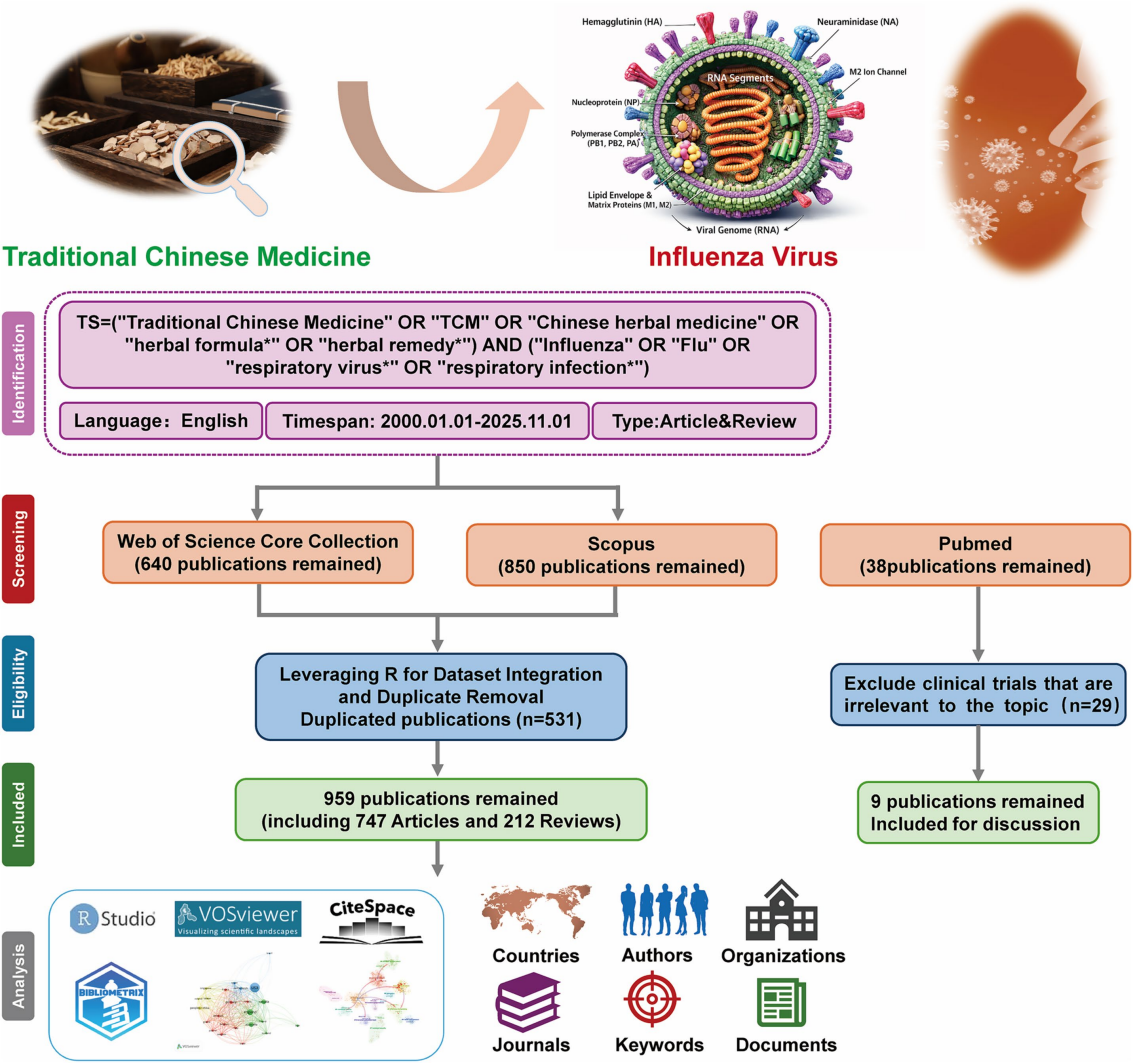
Workflow for literature retrieval and data cleaning in TCM influenza research. This workflow depicts the systematic selection of literature on traditional Chinese medicine (TCM) in influenza research across multiple databases. It traces the process from initial retrieval and deduplication through title/abstract screening and full-text evaluation to final inclusion, ensuring scientific rigor and reproducibility.

This study conducted a systematic literature search across three databases: the Web of Science Core Collection (WoSCC), Scopus, and PubMed. The aim was to identify relevant research publications and clinical trials related to the use of traditional Chinese medicine (TCM) in the treatment of influenza, to support bibliometric analysis. Original articles and reviews were retrieved from WoSCC and Scopus, while PubMed was queried for closely related clinical trials. A comprehensive list of the search strategies, including the complete search strings, is provided in Supplementary Table 1. To ensure data integrity and prevent duplication, we employed a combination of automated de-duplication and manual checks. This approach allowed for the elimination of redundant entries (Figure 1). After curating the literature on traditional Chinese medicine-based influenza research, we applied the same standardized data-cleaning workflow to publications on Western antiviral drugs to support subsequent bibliometric analyses and enable robust cross-domain comparisons (Supplementary Figure 1 and Supplementary Table 2). The final search date across all databases was November 1, 2025. The search, de-duplication, and initial data processing were independently conducted by two researchers, and any discrepancies were resolved through discussion and collaboration.

### 2.2 Data analysis

Several leading bibliometric analysis tools were employed to process and analyze the retrieved data, including CiteSpace (version 6.4.R1), VOSviewer (version 1.6.20), and the R-Bibliometrix package (version 5.0) within R-Studio, for visualization and comprehensive analysis (Synnestvedt et al., 2005; Arruda et al., 2022). Additionally, Microsoft Excel 2024 was used to generate bar charts and line graphs. Bibliometrix is a comprehensive analysis package based on the R programming language, equipped with multi-dimensional analysis capabilities and visualization functions. This tool supports cross-database data integration and can handle metadata from multiple sources of literature in a unified manner (Bamahel et al., 2025). In this study, Bibliometrix (version 5.0) was used to merge data from WoSCC and Scopus, and key bibliometric indicators for each database were manually reviewed and recorded (Figure 1).

Furthermore, based on the PubMed search results, we summarized the characteristics of relevant clinical trials, including the herbal components used in the formulations, the study periods, and the conclusions drawn. This provides supplementary insights into the clinical aspects of TCM in influenza treatment research. It is important to note that only studies explicitly employing Chinese herbal components in their interventions were included in the analysis.

### 2.3 Standardization of author, institution, and keyword data

To address variations in author names, inconsistent capitalization, and formatting discrepancies, we standardized author information, institutions, and keywords in the raw data. Due to the lack of unique author identifiers (e.g., ORCID), some author nodes may still have minor duplication or erroneous merging. Consequently, the results and conclusions are based on macro-level collaboration patterns. To improve consistency in the institution collaboration network, Bibliometrix’s “Institution Name Disambiguation” feature was used to merge variant names for the same institution.

In keyword processing, researchers manually standardized synonyms and near-synonyms and removed generic terms unrelated to the research hotspots to maintain focus on the core themes.

## 3 Results

### 3.1 Overview of publication trends

Between 2000 and 2025, a total of 640 and 850 publications were retrieved from the Web of Science Core Collection (WoSCC) and Scopus databases, respectively. After de-duplication and consolidation, 959 unique publications were included in the analysis, sourced from 407 distinct journals and publications. The annual average growth rate of literature in this field was 16.94% (combined WOSCC + Scopus), with WOSCC contributing an 18.39% increase and Scopus showing a 16.45% growth rate, reflecting the rapid development of research on traditional Chinese medicine (TCM) in influenza treatment. The average age of the publications was 6.16 years, indicating that the research output is relatively recent, with an average of 27.4 citations per article (for the combined dataset). Notably, publications from the Scopus database had a higher average citation count (30.12), which may be attributed to the inclusion of a greater proportion of review articles (Table 1).

**Table 1:** Comparison of overall statistics between databases (WOSCC, SCOPUS, WOSCC + SCOPUS).

Description	WOSSC	Scopus	WOSSC + Scopus
Timespan	2000:2025	2000:2025	2000:2025
Sources (journals, books, etc.)	247	366	407
Documents	640	850	959
Annual growth rate %	18.39	16.45	16.94
Document average age	5.51	6.27	6.16
Average citations per doc	23.38	30.12	27.4
Keywords plus (ID)	1,655	11,299	7,959
Author’s keywords (DE)	1,788	2,325	2,567
Authors	4,011	4,871	2,915
Authors of single-authored docs	7	24	27
Single-authored docs	7	24	27
Co-authors per doc	7.71	7.12	7.11
International co-authorships %	15.34	18	10.23
Article	541	659	747
Review	99	191	212

Compares the overall statistics of WOSCC, SCOPUS, and their combined datasets, highlighting key metrics such as publication volume, citation counts, and impact factor.

In the keyword analysis, a total of 7,959 *Keywords Plus* and 2,567 *Author’s Keywords* were extracted, underscoring the breadth and diversity of research within this field. On the author level, 2,915 distinct authors contributed to the publications, with a minimal number of single-author papers (only 27), suggesting that the majority of publications are collaborative in nature. The average number of co-authors per paper was 7.11, reflecting a high degree of team-based collaboration. International collaboration accounted for 10.23% of the total, which remains relatively low. In terms of publication types, research articles (747 papers, 77.9%) predominated, while review articles (212 papers, 22.1%) also constituted a significant portion of the literature (Table 1).

### 3.2 Publication trends and geographical distribution

**Figure 2:**
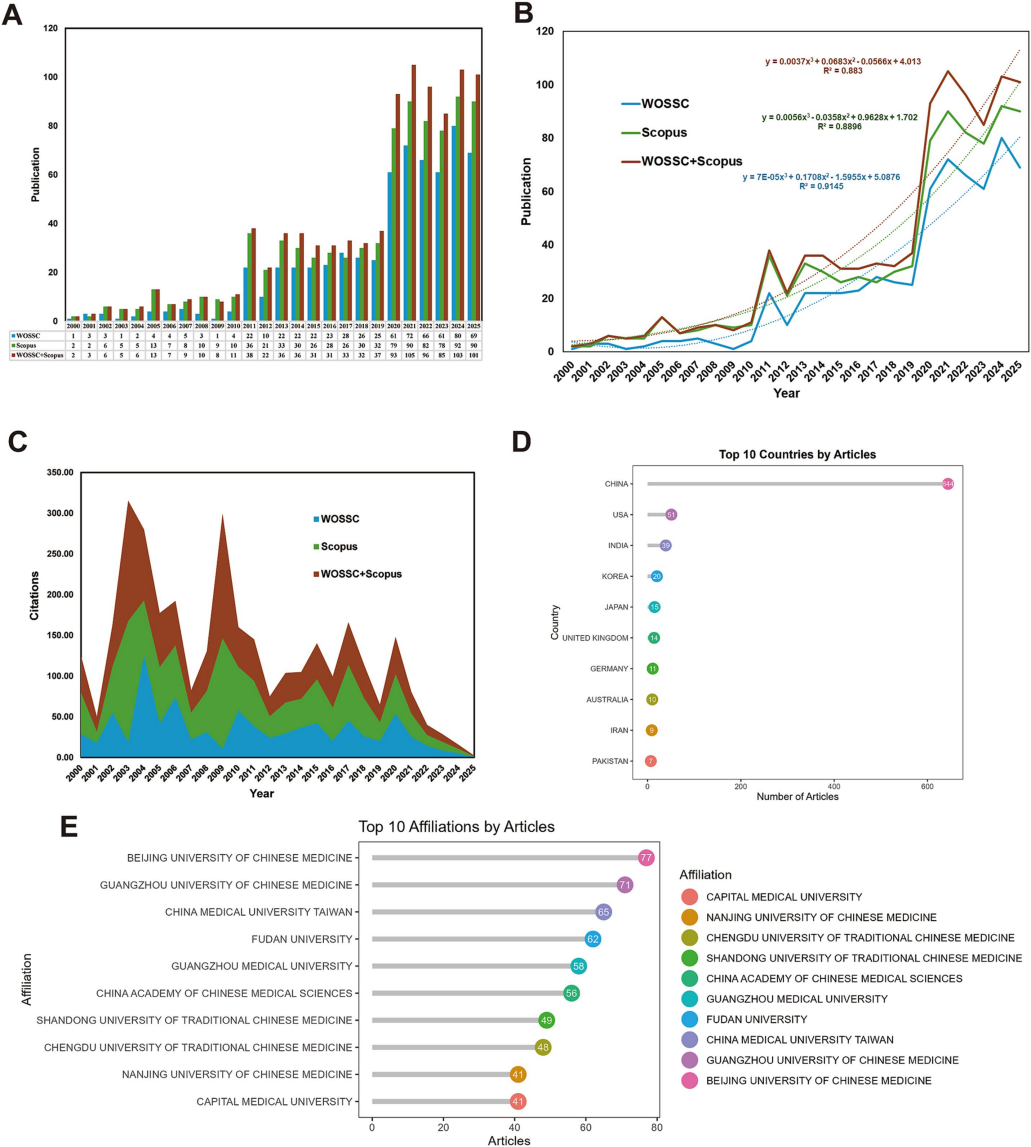
Publication trends and geographical distribution. **(A)** Annual publication trends: displays the annual publication trends in the field of traditional Chinese medicine (TCM) for influenza treatment, based on data from WOSCC, SCOPUS, and their combined datasets. **(B)** Fitted publication volume curve: illustrates the fitted curve representing publication volume over time for WOSCC, SCOPUS, and their merged dataset. **(C)** Annual citation trends: depicts the annual citation trends for TCM-related influenza research across WOSCC, SCOPUS, and combined data. **(D)** Top 10 countries by publication volume: ranks the top 10 countries by publication volume in TCM for influenza research, based on WOSCC and SCOPUS data. **(E)** Top 10 institutions by publication volume: ranks the top 10 institutions by publication volume in TCM for influenza research, derived from WOSCC and SCOPUS data.

One of the most direct ways to assess the evolution of a discipline or field is by observing the changes in annual publication volume. We systematically analyzed the publication trends related to traditional Chinese medicine (TCM) in the treatment of influenza using data from the Web of Science Core Collection (WoSCC), Scopus, and the combined dataset of both sources. Overall, the number of relevant studies has shown a steady increase since 2000, with a significant acceleration in growth from 2010 onwards. Following 2019, a dramatic surge in publications occurred, peaking in 2021 with a total of 105 publications from WoSCC and Scopus combined. This sharp increase reflects the heightened global focus on TCM interventions for respiratory infections in the context of the COVID-19 pandemic. Although there has been some fluctuation in recent years, the overall trend remains elevated, indicating a sustained high level of interest in TCM for influenza treatment (Figure 2A).

We further fitted the annual publication data to a time-series curve, which revealed a clear nonlinear growth trend across all datasets (WoSCC, Scopus, and the combined dataset). The composite curve from the combined data (red solid line) illustrates a notable acceleration in growth after 2019. Polynomial regression analysis (*R*^2^ = 0.883) demonstrated a high level of fit, reinforcing the observation that the research investment and output in this field have both intensified (Figure 2B).

Regarding the cumulative citation distribution of publications by year, early papers (such as those from 2003 and 2009) received higher citation counts due to their relevance to key epidemic events, such as SARS and the H1N1 influenza pandemic. This resulted in two distinct citation peaks (Figure 2C and Table 2).

**Table 2:** Annual citation analysis by database (WOSCC, SCOPUS, WOSCC + SCOPUS).

Year	WOSSC mean TC per article	Scopus mean TC per article	WOSSC + Scopus mean TC per article
2000	29.00	50.50	45.00
2001	18.00	14.00	18.00
2002	55.67	56.33	51.00
2003	19.00	148.80	147.40
2004	126.00	67.00	86.83
2005	42.00	69.23	66.08
2006	74.00	63.86	54.29
2007	22.60	32.75	27.00
2008	31.33	50.70	48.40
2009	11.00	135.56	152.12
2010	58.00	53.50	48.55
2011	38.55	55.67	50.87
2012	24.40	26.43	23.91
2013	29.55	38.09	36.19
2014	36.82	35.40	32.81
2015	42.32	53.92	43.90
2016	20.35	41.00	37.87
2017	45.79	68.23	51.79
2018	26.00	46.90	40.72
2019	20.40	22.97	21.46
2020	54.25	48.19	45.25
2021	25.53	28.46	26.48
2022	14.33	13.29	12.38
2023	8.59	10.50	9.25
2024	5.18	5.48	4.90
2025	0.78	0.70	0.70

Presents the annual citation trends for publications in TCM and influenza research, based on WOSCC, SCOPUS, and their combined dataset.

On a global scale, China leads the publication count by a wide margin, with 644 papers, underscoring its dominant role in the field of TCM-based influenza research. The United States ranks second with 51 papers, followed by India, South Korea, Japan, and other countries, indicating that this area of research is increasingly gaining international attention (Figure 2D). However, despite having far fewer publications than China, countries like the United States, the United Kingdom, and Germany exhibit a high proportion of multi-country publications (MCP), indicating that their research is heavily reliant on international collaboration (Table 3).

**Table 3:** Top 10 countries by publication volume (WOSCC + SCOPUS).

Country	Articles	Articles %	SCP	MCP	MCP %
CHINA	644	67.2	587	57	9
USA	51	5.3	37	14	27.5
INDIA	39	4.1	38	1	2.6
KOREA	20	2.1	17	3	15
JAPAN	15	1.6	15	0	0
UNITED KINGDOM	14	1.5	11	3	21.4
GERMANY	11	1.1	8	3	27.3
AUSTRALIA	10	1	9	1	10
IRAN	9	0.9	9	0	0
PAKISTAN	7	0.7	7	0	0

Ranks the top 10 countries by publication volume in the TCM-influenza research field.

Among the top 10 research institutions by publication volume, Beijing University of Chinese Medicine leads with 77 papers, followed by Guangzhou University of Chinese Medicine (71 papers) and China Medical University, Taiwan (65 papers). This reflects the central role of Chinese traditional medicine universities in this field (Figure 2E). Additionally, we have compiled the Research Organization Reference ID (RORID) for the top 10 institutions to eliminate any potential ambiguities (Table 4).

**Table 4:** Top 10 institutions by publication volume (WOSCC + SCOPUS).

Affiliation	ROR ID	Articles
BEIJING UNIVERSITY OF CHINESE MEDICINE	05damtm70	77
GUANGZHOU UNIVERSITY OF CHINESE MEDICINE	03qb7bg95	71
CHINA MEDICAL UNIVERSITY TAIWAN	00v408z34	65
FUDAN UNIVERSITY	013q1eq08	62
GUANGZHOU MEDICAL UNIVERSITY	00zat6v61	58
CHINA ACADEMY OF CHINESE MEDICAL SCIENCES	042pgcv68	56
SHANDONG UNIVERSITY OF TRADITIONAL CHINESE MEDICINE	0523y5c19	49
CHENGDU UNIVERSITY OF TRADITIONAL CHINESE MEDICINE	00pcrz470	48
CAPITAL MEDICAL UNIVERSITY	013xs5b60	41
NANJING UNIVERSITY OF CHINESE MEDICINE	04523zj19	41

Ranks the top 10 institutions by publication volume in the TCM-influenza research field.

### 3.3 Author, institution, and country productivity analysis

**Figure 3:**
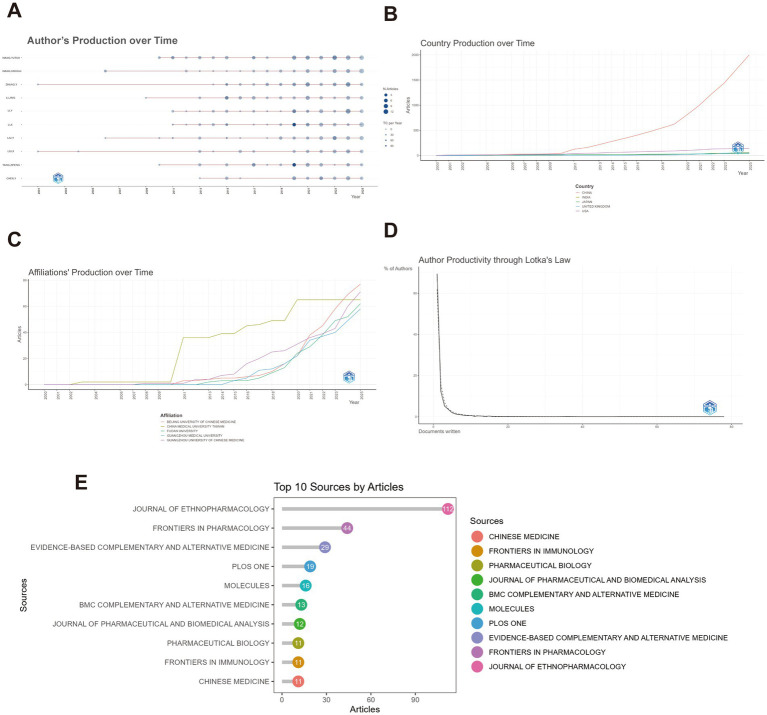
Author, institution, and country productivity analysis. **(A)** Annual author productivity: presents the yearly output of authors in TCM-influenza research, using data from WOSCC and SCOPUS. **(B)** Annual country productivity: shows the annual publication output of countries in the field of TCM for influenza treatment. **(C)** Annual institution productivity: depicts the yearly publication output of institutions in the TCM-influenza research area. **(D)** Author productivity following Lotka’s law: displays the distribution of author productivity in the field of TCM for influenza, illustrating the skewness in publication frequency based on Lotka’s law.

Although the field boasts a large number of researchers, publication output is relatively concentrated among a few high-producing authors. Authors such as *WANG*, *YUTAO*, *ZHANG*, *Y*., and *YANG*, *ZIFENG* have consistently maintained high research output over an extended period, highlighting their significant contributions and leadership in the field (Figure 3A). Analysis based on Lotka’s law reveals that approximately 80% of authors have published only one paper, while fewer than 10% have authored three or more articles, illustrating the high degree of imbalance in scientific output (Figure 3B). This “long-tail distribution” phenomenon suggests that while a few core experts lead the research frontier, much of the work remains exploratory, underscoring the need to establish more effective collaborative mechanisms to enhance overall research efficiency. Furthermore, we used the R package to calculate the top 10 authors ranked by academic contribution indicators (such as the H-index) (Table 5).

**Table 5:** Top 10 authors by H-index (WOSCC + SCOPUS).

Author	h_index	g_index	m_index	TC	NP	PY_start
WANG YUTAO	23	42	1.438	1,915	78	2010
LI Y	20	30	1.333	1,004	50	2011
LI, JING	19	32	1.118	1,122	50	2009
YANG ZIFENG	19	38	1.188	1,514	40	2010
WANG XINHUA	17	31	0.85	1,018	52	2006
ZHANG Y	17	35	0.68	1,268	52	2001
LIU Y	16	36	0.8	1,299	46	2006
LI X	15	42	1	1,772	48	2011
LI Z	14	28	0.56	825	32	2001
WANG Z	14	30	0.933	914	34	2011

Ranks the top 10 authors by their H-index in the TCM-influenza research field.

At the institutional level, Beijing University of Chinese Medicine has experienced steady growth in publications since 2011, with a significant breakthrough in 2020, positioning it as one of the most active research centers in this field. Similarly, China Medical University, Taiwan, has demonstrated strong research momentum since 2010, reflecting the collaborative advancement of TCM research across the Taiwan Strait (Figure 3C).

At the national level, China is the undisputed leader in this field, with its publication output surpassing that of other countries since 2010. While the United States, Japan, India, the United Kingdom, and other nations have made contributions, their output is relatively small, and their growth momentum remains insufficient. Apart from China, research investment from other countries in this area remains limited, indicating an urgent need to strengthen international collaboration (Figure 3D).

In terms of journal distribution, *Journal of Ethnopharmacology* leads with 112 publications, making it the most influential international journal in this field. It is followed by *Frontiers in Pharmacology* (44 papers), *Evidence-Based Complementary and Alternative Medicine* (29 papers), and others, all of which are prominent journals in the fields of pharmacology and complementary and alternative medicine. The concentration of publications in these journals indicates that TCM-based influenza research is increasingly integrated into mainstream international scientific frameworks, with its findings being disseminated and published in high-impact platforms (Figure 3E). We also calculated the academic metrics and publishing information of high-impact journals in the field of TCM-based influenza research, covering the top 10 international journals with the most publications in this domain (Table 6).

**Table 6:** Top 10 journals by H-index.

Source	h_index	g_index	m_index	TC	NP	PY_start
JOURNAL OF ETHNOPHARMACOLOGY	36	54	1.714	3,411	112	2005
FRONTIERS IN PHARMACOLOGY	16	30	2.667	972	44	2020
EVIDENCE-BASED COMPLEMENTARY AND ALTERNATIVE MEDICINE	14	22	0.875	523	29	2010
PLOS ONE	14	19	0.933	933	19	2011
BMC COMPLEMENTARY AND ALTERNATIVE MEDICINE	12	13	0.632	555	13	2007
AMERICAN JOURNAL OF CHINESE MEDICINE	10	10	0.435	242	10	2003
MOLECULES	10	16	0.625	390	16	2010
FRONTIERS IN IMMUNOLOGY	8	11	0.667	357	11	2014
JOURNAL OF PHARMACEUTICAL AND BIOMEDICAL ANALYSIS	8	12	0.615	288	12	2013
PHARMACEUTICAL BIOLOGY	8	11	0.4	560	11	2006

Ranks the top 10 journals by their H-index in the TCM-influenza research field.

### 3.4 Keyword analysis

**Figure 4:**
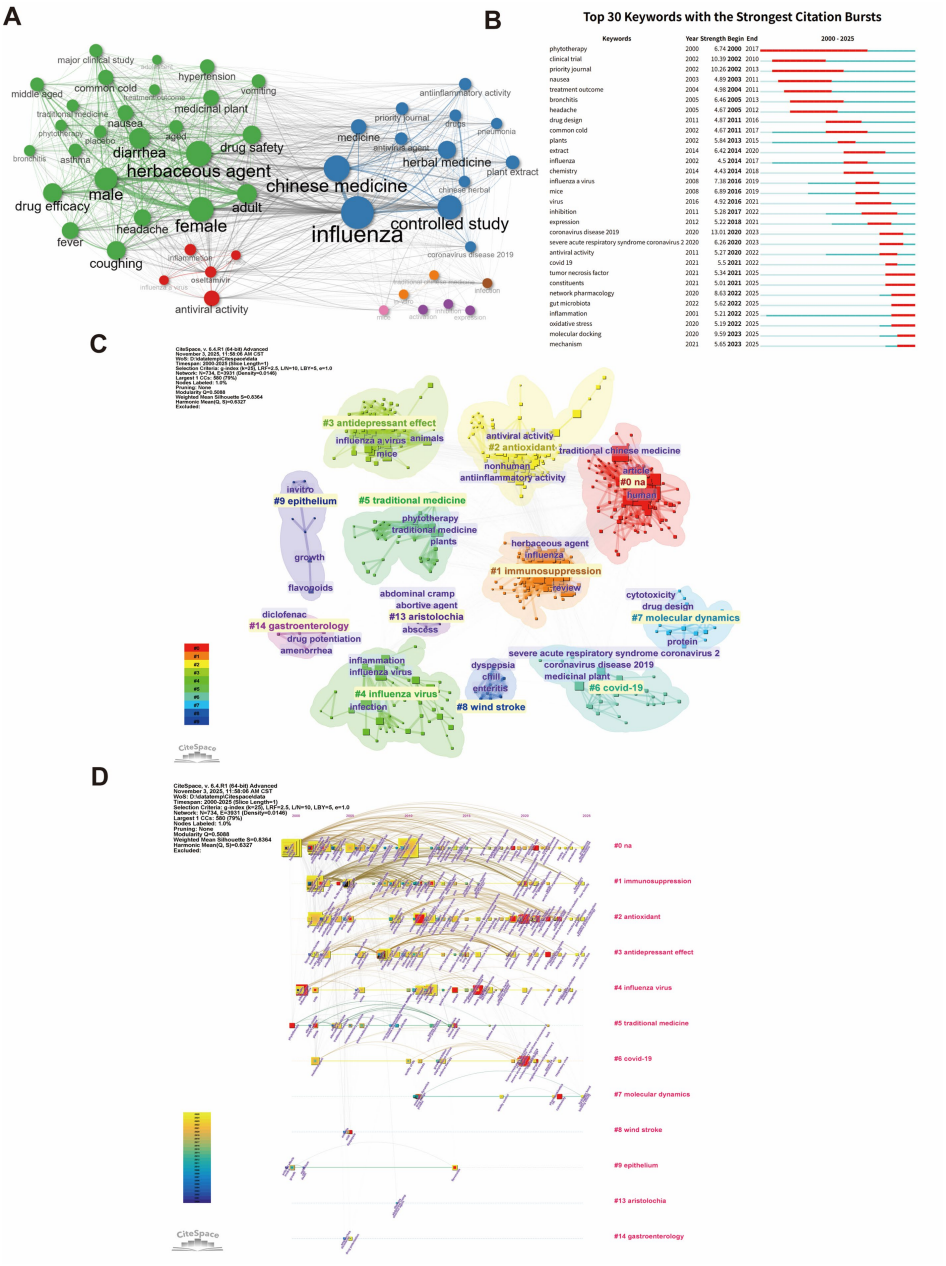
Keyword analysis. **(A)** Co-occurrence network of high-frequency keywords: visualizes the co-occurrence network of high-frequency keywords in TCM and influenza research, highlighting core themes and their interrelations. **(B)** Top 30 keywords by citation burst: ranks the top 30 keywords with the strongest citation bursts, reflecting emerging research trends over time. **(C)** Keyword clustering map: shows the clustering of keywords based on co-occurrence patterns, revealing major research themes and focal areas. **(D)** Keyword timeline: presents the temporal evolution of keywords in TCM-influenza research, identifying emerging and waning topics.

A meticulous analysis of keywords constitutes a vital component of bibliometric methodology. Using R-Bibliometrix, we visualized keyword co-occurrence in publications on traditional Chinese medicine (TCM) for influenza treatment from 2000 to 2025. Node size reflects keyword frequency, while line thickness indicates co-occurrence strength. Central nodes like “influenza” and “Chinese medicine” highlight the focus on TCM interventions. Keywords such as “herbal medicine,” “antiviral activity,” and “phytotherapy” emphasize antiviral mechanisms and clinical efficacy. Terms like “drug safety,” “efficacy,” “fever,” and “cough” reflect research on symptom relief and adverse reactions. Notably, “oseltamivir” appears, signaling growing comparative studies between TCM and Western medicine (Figure 4A). Frequency analysis of the keywords reveals that “influenza” and “traditional Chinese medicine” are at the core of the research, with the top 20 most frequent keywords indicating that TCM-based influenza research is primarily focused on antiviral activity, inflammation regulation, animal studies, and comparisons with oseltamivir. This body of research is also significantly influenced by the COVID-19 pandemic (Table 7).

**Table 7:** Top 20 high-frequency keywords.

Terms	Frequency
influenza	213
traditional Chinese medicine	200
influenza virus	143
Chinese medicine	126
controlled study	106
COVID-19	96
antiviral activity	94
herbaceous agent	93
female	86
herbal medicine	82
infection	81
inflammation	81
male	77
medicine	72
*in-vitro*	64
adult	62
oseltamivir	62
drug efficacy	61
mice	60
diarrhea	57

Lists the top 20 most frequently used keywords in the TCM-influenza research field.

The top 30 keywords with the strongest citation bursts reflect the temporal evolution of research hotspots. For instance, “coronavirus disease 2019” experienced a dramatic burst in 2020 (burst strength = 6.26), continuing until 2023, demonstrating the profound impact of the COVID-19 pandemic on TCM antiviral research. The keyword “COVID-19” peaked in 2021 and then plateaued, although its influence persists to this day. Meanwhile, keywords such as “influenza A virus,” “antiviral activity,” “molecular docking,” and “network pharmacology” have seen frequent bursts in recent years, signaling a shift from traditional empirical methods to modern scientific validation, particularly with notable advancements in network pharmacology and molecular mechanism analysis. Additionally, emerging concepts such as “gut microbiota,” “oxidative stress,” and “immune modulation” underscore the deepening exploration of TCM’s multi-target, multi-pathway regulatory mechanisms (Figure 4B).

We applied the CiteSpace clustering algorithm to categorize the keywords into several thematic clusters, each representing a distinct research direction. The first cluster, labeled Immunosuppression, involves terms such as “herbaceous agent,” reflecting the potential role of traditional Chinese medicine (TCM) in modulating excessive immune responses. Another cluster, antioxidant, incorporates “anti-inflammatory activity,” emphasizing TCM’s ability to scavenge free radicals and mitigate oxidative damage. A third cluster, antidepressant effect, while not directly targeting influenza, suggests TCM’s broader regulatory potential. The Influenza virus cluster, encompassing keywords like “infection,” “inflammation,” and “influenza virus,” focuses on core pathophysiological research. Traditional medicine reflects the ongoing exploration of classic formulations and natural medicinal substances, with associated terms such as “phytotherapy” and “plants.” The COVID-19 cluster illustrates the heightened focus on TCM’s role in combating respiratory viral infections during the COVID-19 pandemic. The molecular dynamics cluster represents the application of computational pharmacology to predict interactions between TCM compounds and viral proteins. Finally, the clusters Wind stroke and Aristolochia indicate that some research has expanded to include other diseases or examine the safety of specific herbs (Figure 4C).

The timeline of keyword evolution provides a clear depiction of the historical development of research themes. Early studies (2000–2010) primarily addressed fundamental topics such as “influenza,” “herbal medicine,” and “clinical trials.” After 2010, keywords related to mechanisms, such as “antiviral activity,” “plant extract,” and “inhibition,” began to rise. Following the outbreak of COVID-19 in 2020, keywords like “covid-19,” “coronavirus disease 2019,” and “SARS-CoV-2” quickly became dominant, facilitating the widespread adoption of modern techniques like “molecular docking” and “network pharmacology.” In recent years, emerging fields such as “gut microbiota,” “immune regulation,” and “metabolomics” have gradually come to the forefront, signaling a growing emphasis on system biology and the holistic regulatory mechanisms of TCM (Figure 4D).

### 3.5 Parallel validation analysis of collaboration among authors, countries, and institutions

This study constructed collaboration networks for authors, countries, and research institutions based on co-occurrence data from the *Web of Science Core Collection* (WOSCC) and *Scopus* databases. A cross-database comparative analysis was conducted to reveal the collaboration patterns in the field of traditional Chinese medicine (TCM) for the treatment of influenza, examining both their consistencies and discrepancies.

**Figure 5:**
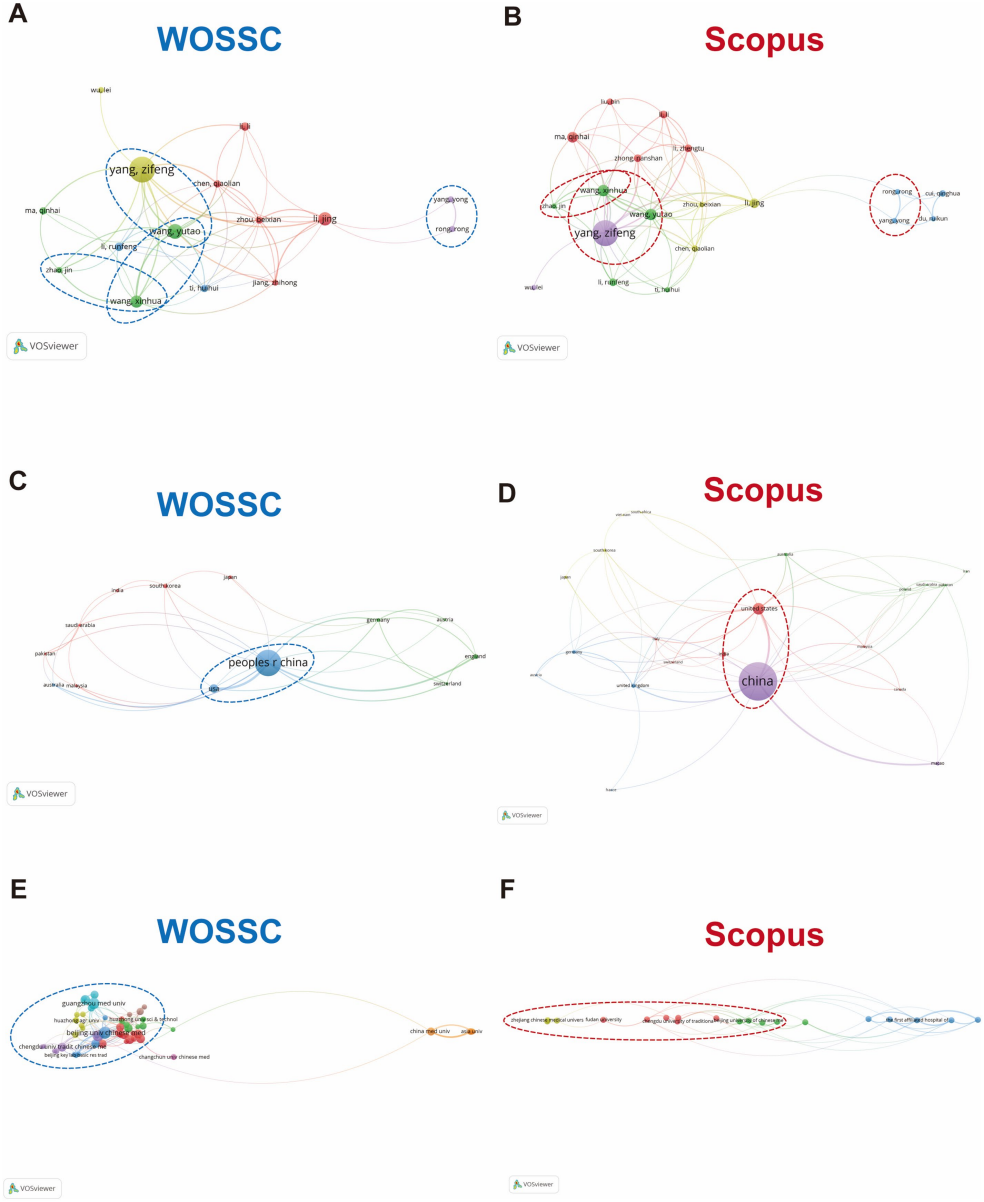
Author, country, and institution collaboration networks. **(A,B)** Author collaboration network: depicts the collaboration network among authors in the TCM-influenza research domain, based on data from WOSCC and SCOPUS. **(C,D)** Country collaboration network: shows the network of collaboration among countries, emphasizing international research partnerships. **(E,F)** Institutional collaboration network: visualizes the collaboration relationships among institutions, highlighting key research hubs in the TCM-influenza field.

In the author collaboration network, Yang, Zifeng holds a central position in both databases, with a large node and high connectivity, signifying that he is one of the most influential scholars in this field. Additionally, researchers such as Wang, Chunhua, Li, Ling, and Chen, Xiaolan show strong associations in both networks, forming a research team centered around Yang, Zifeng. Notably, some authors, such as Rongrong and Cui, Qianhui, exhibit higher collaboration intensity in the Scopus database, suggesting potential database indexing biases or a greater concentration of international collaborations in Scopus. Overall, both databases show a high level of agreement in identifying the core group of authors, further reinforcing the stability of China’s leading position in this research area (Figures 5A,B).

The country-level collaboration network reveals that China occupies a central role in global TCM research collaboration, with varying degrees of cooperation with countries such as the United States, Japan, Australia, and the United Kingdom. However, the overall collaboration network remains somewhat loose, with many connections being relatively weak. This pattern is also reflected in Scopus, suggesting that while China is a major producer of research, there is room for improvement in international collaboration, particularly in facilitating multinational clinical trials and mechanistic studies (Figures 5C,D).

The constructed research institution collaboration network further shows that, within WOSCC, top TCM institutions in China, such as Beijing University of Chinese Medicine, Guangzhou University of Chinese Medicine, Chengdu University of Traditional Chinese Medicine, and Shanghai University of Traditional Chinese Medicine, have formed a close-knit “Chinese TCM University Alliance,” with frequent collaboration among them, resulting in a highly cohesive research cluster (Figure 5E). In contrast, in Scopus, although Chinese institutions still dominate, the collaboration model is more fragmented, with independent nodes emerging from universities such as Zhejiang Chinese Medical University, Shanghai University of Traditional Chinese Medicine, and Changchun University of Traditional Chinese Medicine. Additionally, some institutions, such as the Chengdu University of Traditional Chinese Medicine Affiliated Hospital, have formed cross-regional collaboration chains with foreign hospitals (Figure 5F). This discrepancy suggests that Scopus includes more results from local universities and affiliated hospitals, highlighting the active participation of grassroots medical institutions, whereas WOSCC focuses more on capturing high-level collaborations within large, comprehensive research institutions.

WOSCC and Scopus show strong consistency in author, country, and institution collaboration networks, particularly with key figures (e.g., Yang Zifeng), leading countries (e.g., China), and institutions (e.g., Beijing University of Chinese Medicine, Guangzhou University of Chinese Medicine). These alignments confirm the reliability of the findings. However, Scopus includes more regionally focused and clinically oriented collaborations, while WOSCC highlights high-quality, high-impact research teams. This parallel validation strengthens the conclusions and provides insights for a unified TCM research evaluation system.

### 3.6 MCA analysis and co-citation network analysis

**Figure 6:**
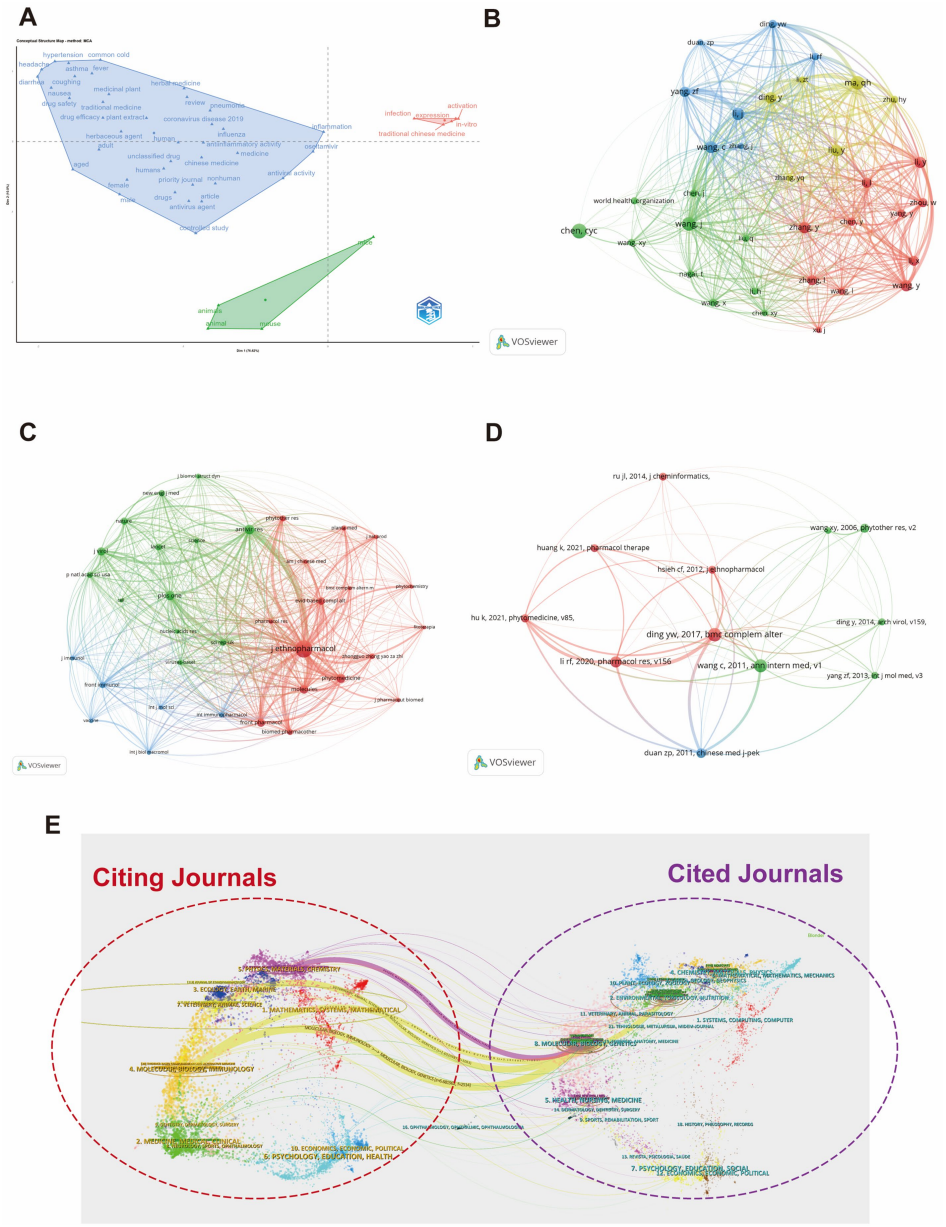
Multiple correspondence analysis (MCA) and co-citation network analysis. **(A)** Multiple correspondence analysis (MCA): displays the results of multiple correspondence analysis, visualizing the relationships between authors, institutions, and keywords in TCM-influenza research. **(B)** Author co-citation network: illustrates the co-citation network of authors, revealing the strength and frequency of co-citations. **(C)** Journal co-citation network: presents the co-citation relationships among journals, identifying the leading journals in TCM and influenza research. **(D)** Literature co-citation network: shows the co-citation network of influential publications, highlighting key papers in the field. **(E)** Dual map overlay analysis: displays a dual-map overlay analysis, revealing academic interactions and interdisciplinary connections between cited and citing journals in the TCM-influenza research domain.

Using multiple correspondence analysis (MCA), we visualized research themes in a two-dimensional space based on keywords, authors, and institutions (Figure 6A). The analysis identified three main clusters: the upper-left region focuses on clinical symptoms like “fever,” “cough,” and “nausea,” reflecting TCM’s approach to managing respiratory symptoms; the upper-right region highlights antiviral mechanisms and molecular biology, underscoring the shift towards microscopic pharmacological studies; and the lower green region emphasizes the role of animal models in preclinical research, bridging traditional practices with modern scientific validation. The author co-citation network shows four core clusters, with influential authors such as Li, Y., Wang, Y., and Zhou, W., while the World Health Organization (WHO) stands out as a significant non-personal node, indicating global attention to TCM in influenza research (Figure 6B). The journal co-citation network reveals key academic journals like Journal of Ethnopharmacology and Phytomedicine as central platforms for TCM research, with Journal of Ethnopharmacology playing a pivotal role in advancing TCM-based influenza research globally (Figure 6C).

The document co-citation network uncovers the citation relationships between the most influential representative works. Centered around Ding Y. W. (2017), a pivotal paper that connects multiple research directions, this document stands out for its substantial impact (Ding et al., 2017). The red cluster (e.g., Li R. F., 2020, Hu K., 2021) focuses on modern pharmacological mechanisms of TCM and natural products (Runfeng et al., 2020; Hu et al., 2021), the green cluster (e.g., Wang X. Y, 2006, Wang C., 2011) highlights antiviral and immune regulation studies (Wang et al., 2011), and the blue cluster (e.g., Duan Z. P, 2011) leans more towards integrating TCM theory with clinical applications (Duan et al., 2011) (Figure 6D). Overall, the field has evolved from early antiviral explorations to more systematic research incorporating network pharmacology and multi-target mechanism analyses, indicating a deep integration of traditional TCM theories with modern scientific methods.

The dual-map overlay analysis illustrates the flow of knowledge between “cited journals” and “citing journals,” highlighting the interdisciplinary nature of the research field (Figure 6E). The red dashed circle on the left represents the citing journals (research domains), while the purple dashed circle on the right represents the cited journals (knowledge sources). The overlap reveals key knowledge dissemination pathways, with molecular biology, immunology, and genetics acting as central hubs connecting both circles. This indicates that life sciences play a pivotal role in the field, facilitating the absorption and dissemination of research. Additionally, fields such as medicine, health, and physics show strong bidirectional interactions, reflecting the integration of basic sciences with clinical medicine and public health.

### 3.7 Analysis of theme evolution trends

**Figure 7:**
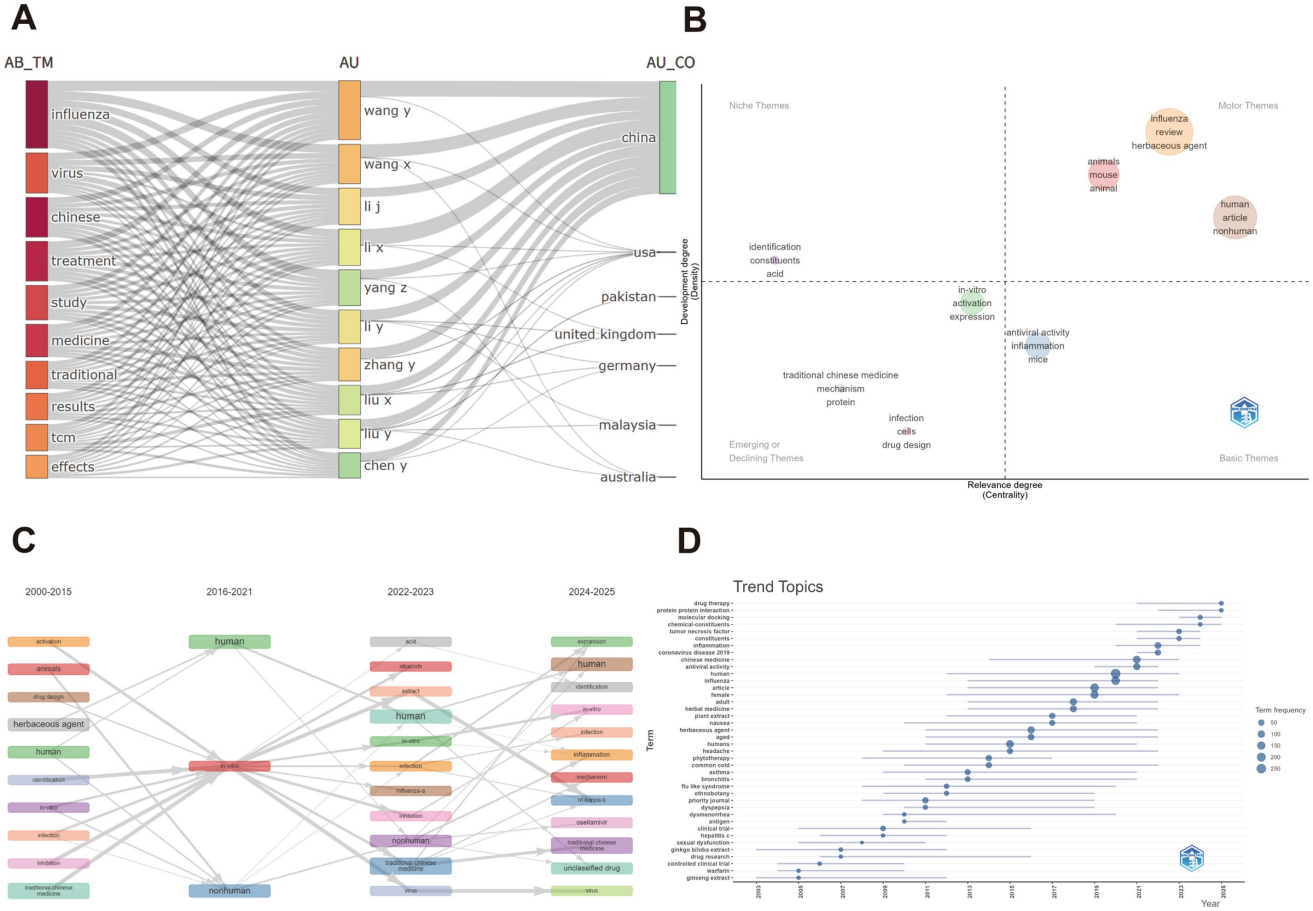
Theme evolution trends analysis. **(A)** Sankey diagram: shows the dynamic relationships between research themes, authors, and countries over time, illustrating the evolution of TCM research in influenza treatment. **(B)** Theme centrality map: visualizes the centrality and activity of major themes in TCM-influenza research, providing insights into emerging research areas. **(C)** Theme evolution map: displays the progression of research themes, indicating shifts in focus within the TCM-influenza domain. **(D)** Theme trend map: presents the trend of key themes over time, identifying areas of rapid growth and decline.

A Sankey diagram was used to visualize the dynamic relationships between “themes,” “authors,” and “countries.” The core themes of the research focus on influenza and traditional Chinese medicine (TCM), with most high-output authors centered on these two topics. Chinese scholars dominate research on “influenza,” “TCM,” and “herbal formulations,” while countries like the United States and the United Kingdom emphasize “drug design,” “mechanisms,” and “inflammation.” Notably, “COVID-19” has emerged as a key theme, particularly among Chinese scholars, reflecting the pandemic’s significant impact on TCM-based antiviral research (Figure 7A).

The thematic centrality map highlights the density (activity level) and centrality (core status) of research themes, helping identify research hotspots and emerging trends. The *x*-axis shows relevance, while the *y*-axis represents development level (Figure 7B). The upper-right quadrant highlights “driving themes” such as herbal formulations, review articles, and animal models, reflecting current research on the antiviral effects of herbal formulations in animal models. The upper-left quadrant represents “niche themes” like compound identification techniques, which, though explored, have not yet become central. The lower-right quadrant contains “fundamental themes” such as antiviral activity and inflammatory response mechanisms, indicating relevance but limited exploration. The lower-left quadrant shows “emerging or declining themes” like the modern transformation of TCM and molecular mechanisms, reflecting lower current activity. Overall, research is primarily focused on herbal formulations and animal models, with future studies needed to explore molecular mechanisms and clinical applications.

The thematic evolution over time can be divided into four stages. From 2000 to 2015, research centered on “traditional Chinese medicine,” “herbal formulations,” and “*in vitro*” experiments, focusing on screening classical formulations and pharmacological activity, addressing the question of “whether TCM is effective.” From 2016 to 2021, new keywords such as “human,” “clinical trial,” and “oseltamivir” emerged, marking a shift towards human interventions and comparative studies, highlighting the integration of TCM and Western medicine. Between 2022 and 2023, keywords like “COVID-19,” “tumor necrosis factor,” and “oxidative stress” broadened the focus to immune regulation and inflammatory pathways. From 2024 to 2025, keywords such as “human,” “inflammation,” “mechanisms,” and “ginseng extract” are expected to dominate, with a focus on individualized treatment, multi-target effects, and the precise use of TCM components (Figure 7C).

The theme trend evolution map shows a steady rise in topics like “influenza,” “herbal medicine,” and “antiviral activity” since 2005, accelerating after 2018. Emerging themes such as “COVID-19,” “immune modulation,” and “network pharmacology” rapidly surged after 2020, marking new research peaks. Specific herbs like “ginseng extract” and “ginkgo leaf extract” have also seen increased focus, reflecting a shift from holistic formula effects to the study of individual active components. Meanwhile, research on basic symptoms like “cold,” “fever,” and “cough” has plateaued, indicating maturity in symptom management, while frontier studies are now increasingly focusing on complex systems such as immune modulation, gut microbiota, and the neuro-immune-endocrine network (Figure 7D).

### 3.8 Citation analysis

**Figure 8:**
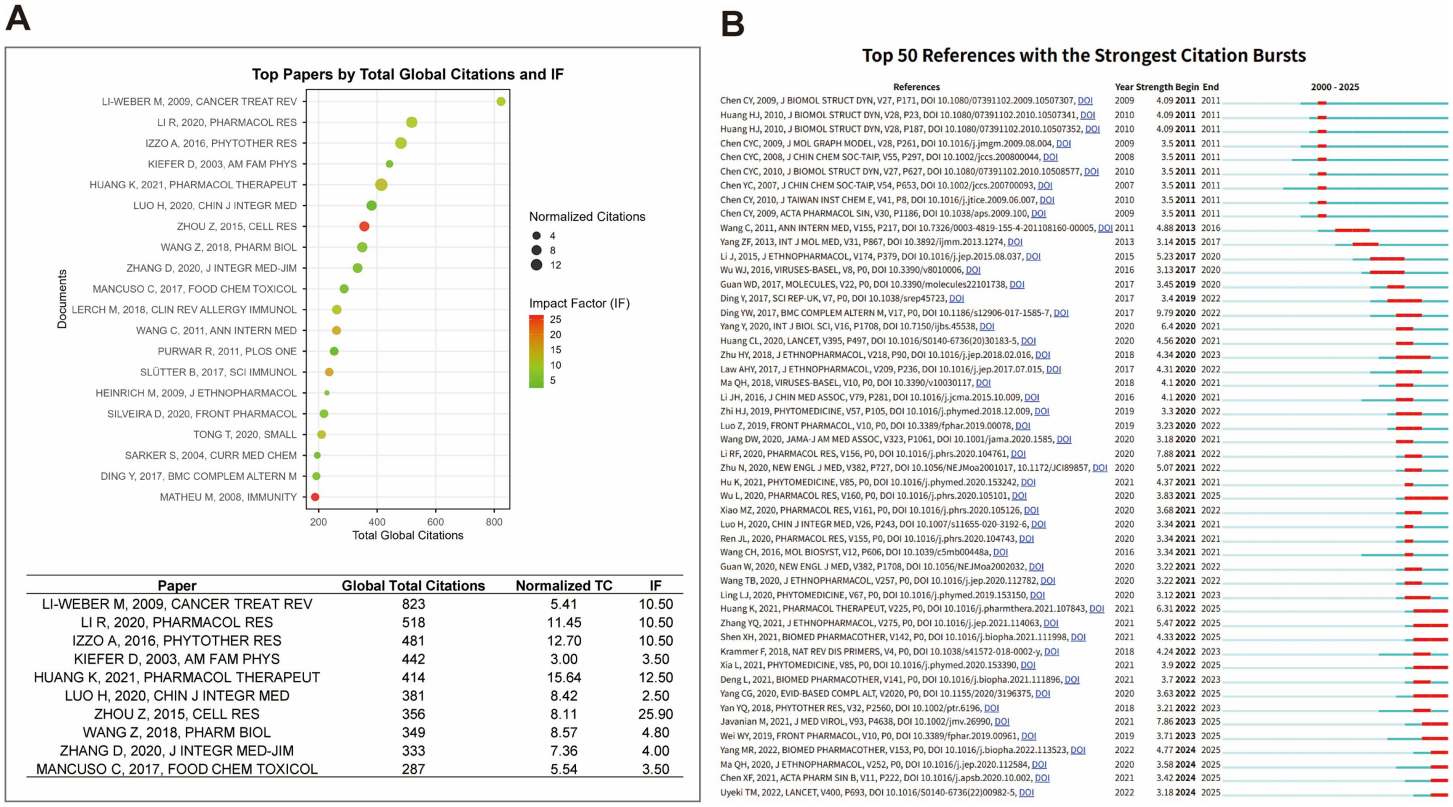
Citation analysis. **(A)** Citation bubble chart of top 20 global cited papers: displays the top 20 most-cited papers in the TCM-influenza research field, with bubble size representing total global citations and impact factor (IF). **(B)** Citation burst analysis of top 50 papers: illustrates the citation burst intensity of the top 50 most influential papers, reflecting the rising trend in citations over time.

We analyzed highly cited literature and citation burst intensity to identify key academic contributions, knowledge dissemination pathways, and evolving trends in traditional Chinese medicine (TCM) for influenza treatment. A bubble chart displays the top 20 most-cited papers from the Web of Science Core Collection (WOSCC) and Scopus, highlighting total citations, normalized citation counts, and journal impact factors (IF). Detailed metrics for the top 10 papers are also included (Figure 8A and Table 8).

**Table 8:** Top 20 cited papers in TCM and influenza research (WOSCC + SCOPUS).

Paper	DOI/PMID	Total citations	TC per year	Normalized TC
LI-WEBER M, 2009, CANCER TREAT REV	10.1016/j.ctrv.2008.09.005	823	48.41	5.41
LI R, 2020, PHARMACOL RES	10.1016/j.phrs.2020.104761	518	86.33	11.45
IZZO A, 2016, PHYTOTHER RES	10.1002/ptr.5591	481	48.10	12.70
KIEFER D, 2003, AM FAM PHYS	14596440	442	19.22	3.00
HUANG K, 2021, PHARMACOL THERAPEUT	10.1016/j.pharmthera.2021.107843	414	82.80	15.64
LUO H, 2020, CHIN J INTEGR MED	10.1007/s11655-020-3192-6	381	63.50	8.42
ZHOU Z, 2015, CELL RES	10.1038/cr.2014.130	356	32.36	8.11
WANG Z, 2018, PHARM BIOL	10.1080/13880209.2018.1492620	349	43.63	8.57
ZHANG D, 2020, J INTEGR MED-JIM	10.1016/j.joim.2020.02.005	333	55.50	7.36
MANCUSO C, 2017, FOOD CHEM TOXICOL	10.1016/j.fct.2017.07.019	287	31.89	5.54
LERCH M, 2018, CLIN REV ALLERGY IMMUNOL	10.1007/s12016-017-8654-z	262	32.75	6.43
WANG C, 2011, ANN INTERN MED	10.7326/0003-4819-155-4-201108160-00005	261	17.40	5.13
PURWAR R, 2011, PLOS ONE	10.1371/journal.pone.0016245	253	16.87	4.97
SLÜTTER B, 2017, SCI IMMUNOL	10.1126/sciimmunol.aag2031	236	26.22	4.56
HEINRICH M, 2009, J ETHNOPHARMACOL	10.1016/j.jep.2009.05.028	228	13.41	1.50
SILVEIRA D, 2020, FRONT PHARMACOL	10.3389/fphar.2020.581840	218	36.33	4.82
TONG T, 2020, SMALL	10.1002/smll.201906206	210	35.00	4.64
SARKER S, 2004, CURR MED CHEM	10.2174/0929867043365189	195	8.86	2.25
DING Y, 2017, BMC COMPLEM ALTERN M	10.1186/s12906-017-1585-7	192	21.33	3.71
MATHEU M, 2008, IMMUNITY	10.1016/j.immuni.2008.07.015	188	10.44	3.88

Presents the top 20 most-cited papers in the TCM-influenza research field, with detailed citation data.

Among these, the review article by LI-WEBER M. (2009), titled “*New Therapeutic Aspects of Flavones: The Anticancer Properties of Scutellaria and Its Main Active Constituents Wogonin, Baicalein, and Baicalin*,” holds the top spot with 823 citations. It has an IF of 10.50 and a normalized citation count of 5.41, reflecting its comprehensive discussion of Scutellaria as an anticancer agent and its molecular mechanisms. Its relevance to influenza is highlighted by its prevention of viral infections and its anti-inflammatory and immune-modulatory properties (Li-Weber, 2009). Similarly, LI R. (2020), in “Lianhuaqingwen Exerts Anti-viral and Anti-inflammatory Activity Against Novel Coronavirus (SARS-CoV-2)”, cited 518 times with an IF of 10.50, explores Lianhuaqingwen’s ability to inhibit SARS-CoV-2 replication *in vitro*, alter viral particle morphology, and exhibit anti-inflammatory effects, suggesting its potential as a novel strategy for controlling COVID-19 (Runfeng et al., 2020). IZZO A. (2016)’s article, “*A Critical Approach to Evaluating Clinical Efficacy, Adverse Events and Drug Interactions of Herbal Remedies*,” cited 481 times with an IF of 12.70, provides a systematic evaluation of clinical evidence for herbal medicines and dietary supplements, assessing the efficacy and safety of several common herbs (Izzo et al., 2016). HUANG K. (2021)’s “*Traditional Chinese Medicine (TCM) in the Treatment of COVID-19 and Other Viral Infections: Efficacies and Mechanisms*,” cited 414 times with an IF of 12.50, examines the application of Lianhuaqingwen capsules (LHQW) and other TCM formulations in the treatment of COVID-19, emphasizing their antiviral and immune-regulatory effects (Huang et al., 2021). Other notable high-citation papers, such as LUO H. (2020)’s “*Can Chinese Medicine Be Used for Prevention of Corona Virus Disease 2019 (COVID-19)? A Review of Historical Classics, Research Evidence and Current Prevention Programs*” and ZHOU Z. (2015)’s “*Honeysuckle-encoded Atypical microRNA2911 Directly Targets Influenza A Viruses*,” reflect the ongoing contributions of Chinese scholars in this field (Zhou et al., 2015) (Figure 8A).

Additionally, we have compiled a list of the top 50 references with the strongest citation bursts over the past 25 years (2000–2025), detailing the authors, journals, years, DOI, and the start and end periods of citation bursts. These references are visualized through red bar charts that illustrate the “burstiness” of the citations. Early burst references (2008–2011), such as Chen C. Y. (2009) and Huang H. J. (2010), marked the introduction of computer-aided drug design (CADD) into TCM research, advancing the scientific process of mechanism exploration (Huang et al., 2010; Chen et al., 2009). Mid-period burst references (2015–2020), such as Yang Z. F. (2013) and Li J. (2015), focused on the regulation of inflammatory pathways, apoptosis mechanisms, and the efficacy evaluation of natural product extracts, reflecting the deepening exploration from macro efficacy to micro mechanisms (Li J. et al., 2015; Yang et al., 2013). Recent burst references (2020–2025), such as Ding Y. (2021), Luo H. (2020), Zhu N. (2020), and Yang T. (2021), primarily emerged in the context of the COVID-19 pandemic. Notably, Zhu N. (2020), although not directly targeting influenza, has been widely cited due to its contribution to COVID-19 treatment protocols, thus enhancing the attention on TCM-based antiviral research (Zhu et al., 2020). References like Chen C. Y. (2009) and Huang H. J. (2010) have shown citation bursts lasting over 3 years, indicating that the theoretical frameworks or technical methods proposed in these works continue to guide subsequent research, frequently being cited (Huang et al., 2010; Chen et al., 2009) (Figure 8B).

### 3.9 Comparative trends in traditional Chinese medicine and Western antiviral research

To systematically assess the academic trajectories of traditional Chinese medicine (TCM) and Western antiviral drugs—represented by oseltamivir, zanamivir, peramivir, baloxavir, and neuraminidase inhibitors—in influenza research, we applied a standardized data-cleaning and analysis workflow to Western antiviral publications following completion of TCM literature preprocessing (Supplementary Figure 2 and Supplementary Table 2).

**Figure 9:**
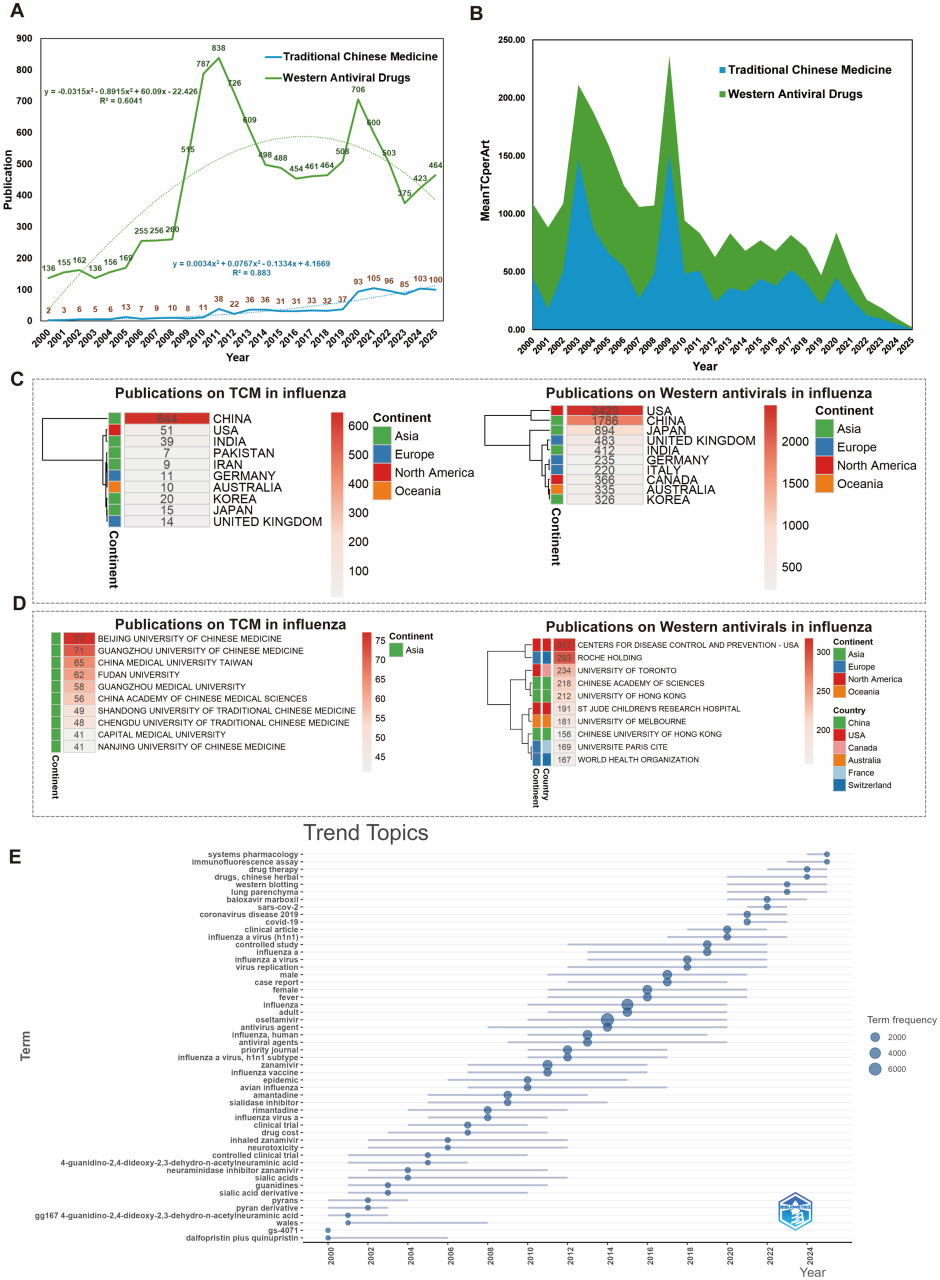
Comparative analysis of TCM and Western antiviral research on influenza (based on combined WOSCC and Scopus data). **(A)** Annual publication trends: displays the yearly publication counts for TCM and Western antiviral studies, with cubic trend lines highlighting temporal growth patterns in both research fields. **(B)** Mean citations per article: illustrates the average citations per article for TCM and Western antiviral research, calculated as total citations divided by publication count, reflecting the relative academic impact of each category. **(C)** Top 10 countries by publication volume: shows the leading countries contributing to TCM and Western antiviral research, ranked by total publication output. **(D)** Top 10 institutions by publication volume: depicts the principal institutions driving research in TCM and Western antiviral studies, ranked according to the number of published articles. **(E)** Evolution of research topics in Western antiviral studies: maps the temporal development of major research topics within Western antiviral influenza studies, highlighting shifts in scientific focus over the past 25 years.

Western antiviral research has experienced a marked expansion since 2000, peaking at 838 publications in 2011, although no consistent growth trend was observed thereafter (*R*^2^ = 0.6041) (Figure 9A). Annual output in this field consistently surpassed that of TCM. By contrast, TCM influenza research emerged later, producing fewer than 15 articles per year from 2000 to 2007. Starting in 2010, TCM publications increased steadily, reaching 105 articles in 2021 before stabilizing. Despite lower absolute numbers, TCM research exhibited a robust average annual growth rate of 16.94%, far exceeding the 5.03% observed for Western antiviral research (Supplementary Table 3).

To account for disparities in publication volume, we evaluated academic influence using mean citations per article (mean TC per article), which provides an objective measure of the scholarly impact of individual studies. Analysis of mean TC per article reveals that TCM research reached citation peaks in 2003 and 2009 (147.40 and 152.12 citations per article, respectively), significantly higher than the corresponding values for Western antiviral research (63.80 and 83.94), indicating periods of pronounced influence (Figure 9B and Supplementary Table 4). In Western antiviral research, the highest mean citation was recorded in 2004 (100.46 citations per article) (Supplementary Table 4).

Geographically, Western antiviral research is concentrated in multiple global centers, led by the United States (2,423 publications), China (1,786), Japan (894), and the United Kingdom (483), with North America and Europe collectively contributing nearly half of the total output (Figure 9C). In contrast, TCM influenza research is heavily regionalized, dominated by China (644 publications) with minor contributions from India (Yin et al., 2017) and Pakistan (Chan and Hui, 2023), reflecting a localized research footprint (Figure 9C). International collaboration is also more prevalent in Western antiviral research (20.13%) than in TCM (10.23%), suggesting greater integration within global scientific networks (Supplementary Table 3). China contributes 67.2% of global TCM publications, yet only 9% involve international collaboration. In Western antiviral research, China ranks second (16.1%), behind the United States (21.8%), but its collaboration rate (15.4%) remains lower than that of Australia (30.7%), Canada (26.0%), and the United Kingdom (25.9%). Notably, despite modest TCM output in the United States (5.3%), over a quarter (27.5%) are international collaborations; in Western antiviral research, the United States functions as a global hub with high productivity and a 20% collaboration rate (Table 3; Supplementary Table 5).

Institutionally, TCM research is led by specialized Chinese universities and institutes, including Beijing University of Chinese Medicine (77 publications), Guangzhou University of Chinese Medicine (Hamazaki et al., 2007), and China Medical University, Taiwan (Wang et al., 2025), reflecting strong disciplinary clustering (Figure 9D). By contrast, Western antiviral research is conducted through a diverse network comprising multinational pharmaceutical companies (e.g., Roche Holding), national public health agencies (e.g., CDC), leading comprehensive universities (e.g., University of Toronto), and international organizations (e.g., WHO), illustrating an interdisciplinary, industry-linked research ecosystem (Figure 9D).

Although Chinese scholars maintain active and stable output in TCM influenza research, their overall scientific impact remains lower than that of Western antiviral researchers (Table 5; Supplementary Table 6). Journal-level comparisons further highlight this disparity: the top 10 journals for TCM influenza research—e.g., Journal of Ethnopharmacology (h-index = 36; total citations = 3,411)—have significantly lower influence than leading Western antiviral journals, such as NEJM (total citations >20,000) and Clinical Infectious Diseases (h-index = 66) (Table 6; Supplementary Table 7).

Thematic analysis reveals that TCM research has gradually shifted from empirical herbal applications to mechanistic studies, with recent adoption of multi-omics, systems pharmacology, and AI-assisted approaches (Figure 7D). By contrast, Western antiviral research has consistently focused on specific drug molecules and their chemical structures (e.g., neuraminidase inhibitors, sialic acids), emphasizing evidence-based studies such as clinical trials and controlled studies. Following the 2009 H1N1 pandemic, research rapidly incorporated public health topics (“influenza A virus (H1N1),” “avian influenza,” “epidemic”), extending to outbreak preparedness and vaccine development. In the 2020s, novel antivirals (e.g., baloxavir marboxil) and the COVID-19 pandemic introduced cross-pathogen research themes (“SARS-CoV-2,” “COVID-19,” “systems pharmacology,” “immunofluorescence assay”), reflecting expansion into integrated multi-pathogen control and systemic drug evaluation (Figure 9E). Overall, TCM research is evolving from empirical practice to mechanistic integration, while Western antiviral research is moving from single-target optimization towards multi-pathogen strategies. In recent years, both domains increasingly converge on interdisciplinary approaches and precision-oriented public health applications.

## 4 Discussion

### 4.1 Discussion based on bibliometric analysis results

Our study indicates a consistent growth in the field since 2000, with a particularly significant rise in research output post-2010, followed by an explosive increase after 2019. This surge in research attention coincides with the global focus on traditional Chinese medicine (TCM) for influenza treatment, especially in the context of emerging viruses, such as SARS-CoV-2, and other respiratory diseases. This trend highlights the increasing global interest in TCM as a potential therapeutic approach for viral infections, reflecting both its historical relevance and emerging applications in the fight against contemporary health crises.

The annual average growth rate of publications (16.94%) and the average age of literature (6.16 years) further underscore the dynamic and evolving nature of research in this field. This phenomenon not only reflects the growing academic interest in TCM for influenza treatment but also signals the continual adaptation and innovation of traditional medicine within modern scientific frameworks. Analysis of highly cited papers and citation bursts reveals that some works have made a significant academic impact, particularly review articles and studies on the integration of TCM with modern pharmaceutical approaches, such as those centered on *Lianhuaqingwen*. These studies have not only deepened the discourse on TCM’s role in combating influenza but also provided fresh perspectives for the theoretical integration of traditional Chinese and Western medicine.

On a global scale, China undeniably dominates the research landscape for TCM-based treatments for influenza. Its output far surpasses that of other countries, highlighting China’s research prowess and international influence in this area. Notable TCM institutions, such as Beijing University of Chinese Medicine and Guangzhou University of Chinese Medicine, have established themselves as key pillars of research, driven by their rich academic heritage and extensive research resources. Despite this leading position, international collaboration remains relatively underdeveloped, particularly in facilitating multinational clinical trials and multicenter research efforts. Strengthening these collaborative mechanisms is crucial for advancing global cooperation in this field.

Further analysis of keywords and co-citation networks reveals a significant shift in the focus of research over recent years. Whereas earlier studies predominantly centered on traditional themes such as “herbal medicines” and “antiviral effects,” the research landscape has expanded to incorporate multidimensional topics like “immune modulation,” “gut microbiota,” and “molecular docking.” This evolution signifies a paradigm shift from broad efficacy studies to more granular, mechanistic investigations, particularly with the support of cutting-edge technologies such as network pharmacology and molecular dynamics. Additionally, the COVID-19 pandemic has undeniably accelerated research in this area, with a noticeable shift in research priorities toward viral immune modulation and the regulation of inflammatory pathways. This trend provides clear direction for the future trajectory of TCM research in the treatment of influenza and other viral infections.

Despite these substantial advancements, several challenges remain that warrant further exploration. For example, many studies continue to focus on evaluating the efficacy of individual herbs or formulations, often overlooking the potential benefits of multi-drug combinations and the optimization of personalized treatment strategies. Moreover, the acceptance and recognition of TCM globally remain limited, particularly in Western countries, where the application of traditional medicine still faces significant barriers. Moving forward, interdisciplinary collaboration, especially the integration of clinical medicine with basic scientific research, will be instrumental in driving the internationalization of TCM-based influenza treatments and fostering global acceptance of these therapeutic approaches.

### 4.2 Mechanism discussion

#### 4.2.1 Inhibitory effects of traditional Chinese medicine components on influenza virus

Traditional Chinese medicine (TCM) treats influenza by enhancing the host’s antiviral immune response and directly inhibiting viral replication. Studies have shown that certain TCM components boost immune function and suppress influenza virus replication through various pathways (Yang et al., 2022; Zhang et al., 2020).

Keishi-ni-eppi-ichi-to (TJS-064) and Hochuekkito (TJ-41) improve survival rates in influenza-infected mice by enhancing immune responses. TJS-064 boosts antiviral functions, while TJ-41 prevents viral replication by increasing type I interferons (IFN) and GM-CSF secretion (Ball et al., 1994; Dan et al., 2013). Similarly, Sanwu Huangqin Decoction (SWHD), through immune modulation and inhibition of influenza virus replication, has shown significant anti-influenza activity, suggesting its potential as an effective complementary therapy in clinical settings (Ma et al., 2018).

Further research revealed that Feiyan Qinghua Decoction (FYQHD) enhances the antiviral response by modulating the gut microbiota and increasing the production of short-chain fatty acids (SCFAs), thereby mitigating the lung and intestinal damage caused by influenza infection. This offers a new theoretical basis for influenza treatment (Dou et al., 2024). Similarly, Sanyang Hezhi Decoction (SYHZ) significantly alleviated influenza symptoms in infected mice by directly inhibiting viral replication, regulating immune responses, and reducing lung injury, suggesting its potential to act through multiple bioactive components (Cheng et al., 2023).

In terms of direct viral inhibition, *Tripterygium wilfordii* (TW) lactone derivatives targeted the influenza virus nucleoprotein (NP) and modulated immune responses, inhibiting influenza A virus replication and revealing substantial potential as a candidate antiviral agent (Jiang et al., 2022). Additionally, Baicalin combined with ribavirin exhibited strong synergistic antiviral effects both *in vitro* and in mice, significantly improving mouse survival rates and alleviating lung inflammation (Chen et al., 2011).

Shuang Huang Lian Injection effectively inhibited H5N1 virus replication and alleviated lung injury, significantly improving mouse survival rates, thereby confirming its efficacy in combating H5N1 virus (Tang et al., 2018). Furthermore, berberine demonstrated potential antiviral therapeutic effects by inhibiting influenza virus replication, reducing lung inflammation, and modulating the TLR7 signaling pathway and immune cell proportions (Yan et al., 2018). Similarly, Honeysuckle’s six bioactive components have been shown to have significant anti-influenza effects (Xie et al., 2022). Compound Yizhihao Granules (CYZH) exhibited broad-spectrum anti-influenza activity by activating the Nrf2/HO-1 pathway, inhibiting influenza A virus (IAV) replication, and protecting cells from oxidative damage, thus providing promising clinical applications for novel antiviral therapies (Yin et al., 2017).

#### 4.2.2 Immunomodulatory effects

The therapeutic effects of traditional Chinese medicine (TCM) in the treatment of influenza extend beyond the enhancement of immune responses; TCM also plays a pivotal role in modulating immune tolerance to prevent excessive immune reactions, thereby alleviating immune-mediated damage (Zhao et al., 2023a). For instance, formulations such as Liu Shen Wan (LSW), along with compounds like α-humulene, apigenin, furanocoumarins, and eucalyptol, exert their effects by inhibiting the TLR4/NF-κB signaling pathway, thereby mitigating the inflammatory responses induced by influenza viruses and enhancing immune tolerance to avoid excessive immune reactions (Chung et al., 2014; Ma et al., 2020; Lv et al., 2025). This immunomodulatory action is crucial for influenza treatment, especially by restoring immune balance through the regulation of the ratios between T cell and B cell subsets.

Studies have demonstrated that the balance between Th1/Th2 and Th17/Treg cell populations is critical to the effectiveness of the immune response (Deng et al., 2016; Shi et al., 2020; Xiao et al., 2023). For example, Xiao-Chai-Hu-Tang (XCHT) has been shown to inhibit influenza virus replication, alleviate cytokine storms, and restore Th17/Treg immune balance, thus effectively protecting mice from combined influenza and *Staphylococcus aureus* infection (Han et al., 2025). Moreover, TCM formulations regulate NLRP3 inflammasome activation, reducing the systemic inflammation caused by influenza. A dual honeysuckle extract formulation has been shown to modulate TNF-mediated IFN/NLRP3 activation, restore immune balance, and alleviate acute lung injury from influenza A virus (Li et al., 2025).

TCM also modulates the immune response in influenza by regulating the gut microbiota. For example, Qin Qiao Xiao Du (QQXD) restores gut balance, enhances carbohydrate metabolism, and regulates cyanide amino acid pathways, inhibiting viral replication, reducing cytokine storms, and protecting mice from influenza-induced pneumonia (Lian et al., 2022). Similarly, *Houttuynia cordata* polysaccharides (HCP) improve intestinal barrier function and regulate gut microbiota composition, reducing lung and intestinal damage caused by H1N1 virus, while exhibiting significant anti-inflammatory effects (Chen et al., 2019).

In summary, TCM regulates immune responses by modulating key immune pathways such as NF-κB, JAK/STAT, and TLR signaling (Fu et al., 2018; Wu et al., 2025; Liang et al., 2024; Lu et al., 2025; Zheng et al., 2021). Exploring TCM’s immunomodulatory mechanisms in influenza treatment provides valuable insights for expanding its use and supports the development of new antiviral therapies.

#### 4.2.3 Anti-inflammatory effects and regulation of oxidative stress response

Astragalus polysaccharides (APS), a key component of traditional Chinese Medicine (TCM), alleviate influenza-induced inflammation by modulating immune pathways. As an immunological adjuvant, APS enhances the efficacy of influenza and SARS-CoV-2 vaccines, boosting antibody production, survival rates, and reducing weight loss, while promoting sustained immune responses (Zhao et al., 2023b). Additionally, TCM has been found to attenuate excessive immune responses by downregulating the expression of various pro-inflammatory cytokines such as TNF-α, IL-6, and IL-1β, thereby reducing tissue damage caused by influenza virus infection (Zhao et al., 2021). For example, *Lianhua Qingwen* has demonstrated significant anti-inflammatory effects by promoting the infiltration and polarization of M2 macrophages, alleviating acute lung injury, and suppressing inflammation (Li et al., 2024).

In terms of oxidative stress, influenza virus infection has been shown to induce substantial oxidative damage through the generation of reactive oxygen species (ROS), which exacerbates the burden on the immune system (Deng et al., 2024). For instance, Epigallocatechin gallate (EGCG), a polyphenol from green tea, inhibits influenza A virus replication and reduces ROS levels, significantly improving survival rates and alleviating lung injury in infected mice, highlighting its potential as a novel preventive treatment for influenza (Ling et al., 2012). Moreover, Japanese herbal medicine such as *Hochuekkito* (HKT) has been shown to enhance the body’s defense against influenza by activating mitochondrial and glycolytic metabolism, thereby maintaining metabolic homeostasis in cells infected by influenza A virus (Takanashi et al., 2017).

Classical TCM formulations like Xiao-Chai-Hu-Tang (XCHT) combine antiviral and anti-inflammatory effects, inhibiting both influenza virus and *Staphylococcus aureus* replication, while restoring Th17/Treg immune balance to protect against dual infection (Han et al., 2025).

Most studies on TCM’s anti-inflammatory and antioxidant effects are still in basic and animal research. Large-scale clinical trials are needed to confirm their efficacy and safety. Integrating systems pharmacology and multi-omics can better elucidate TCM’s multitarget effects, supporting personalized treatments and the development of new antiviral agents.

#### 4.2.4 Application of network pharmacology in the treatment of influenza with traditional Chinese medicine

The multifaceted, multitarget nature of traditional Chinese medicine (TCM) makes its therapeutic mechanisms challenging to explain through single-target models. Network pharmacology, by linking drugs, targets, and diseases, uncovers the synergistic multitarget effects of TCM in treating influenza, offering new theoretical support for its clinical application (Nogales et al., 2022). Studies indicate that network pharmacology operates through multiple pathways, including antiviral effects, immune modulation, inflammation inhibition, and the restoration of immune balance, all contributing to the therapeutic potential against influenza (Huang et al., 2023; Tang et al., 2022).

For instance, *Tianlong tea* (TLC) and its major component, baicalin (BCL), exert significant antiviral effects by modulating T-cell receptor signaling pathways, inhibiting influenza virus replication, and mitigating inflammation, thereby demonstrating considerable potential in flu treatment (Wang et al., 2025). Similarly, the active constituents and targets of *Gui Zhi Granules* were screened through TCMSP and STITCH databases, with their primary mechanisms being antiviral and anti-inflammatory effects targeting key proteins such as JUN, TNF-α, and RELA, which help alleviate symptoms of influenza (Ling et al., 2023). Furthermore, *Xiaohu Guizhi Decoction* (CGD), as a complex TCM formulation, exerts a comprehensive therapeutic effect by engaging multiple signaling pathways, showcasing remarkable anti-influenza activity (Feng et al., 2025).

Network pharmacology, coupled with gut microbiota analysis, further reveals that *Xuanbai Chengqi Decoction* (XBCQ) modulates immune injury and regulates the gut microbiota while protecting the lung and intestine from damage via pathways such as TNF, TCR, and NF-κB. This provides strong evidence for its potential in treating viral pneumonia (Huo et al., 2022). Additionally, research indicates that compounds such as platycodin D and luteolin from *Platycodonis Radix* may exert anti-influenza effects by modulating the TNF, IL-17 signaling pathways, and cell death-related pathways (Feng et al., 2024). These findings establish a new theoretical foundation for TCM in treating influenza, supporting its clinical use. Combining network pharmacology with big data aids in identifying key targets and pathways, advancing novel TCM-based therapies. Integrating omics technologies will further enhance TCM’s role in influenza treatment.

### 4.3 Discussion on clinical trials

In recent years, the application of traditional Chinese medicine (TCM) in influenza treatment has gained increasing attention. A structured search of the PubMed database yielded 38 relevant studies, from which 9 clinical trials on influenza treatment were selected after excluding 29 unrelated studies (Table 9).

**Table 9:** Summary of clinical trials on TCM for influenza based on PubMed database.

Year	Research drug/Formulation	Study conclusion	PMID
2007	Hochu-ekki-to (HET)	This study found that HET did not significantly improve the antibody titers post-influenza vaccination, suggesting that the adjuvant effect of some Chinese medicines may be limited	16644196
2013	Jinhua Qinggan Granules (JHG)	In patients with wind-heat influenced febrile syndrome (WHAFS) influenza, low-dose JHG demonstrated significant advantages in reducing fever duration and alleviating symptoms, with no adverse reactions observed	24517059
2015	Banlangen Granules (BLG)	In seasonal influenza treatment, BLG was comparable to oseltamivir in efficacy, particularly in alleviating fever and cough symptoms	25873046
2015	Chima Qingwen Decoction	In clinical trials with patients suffering from mild H1N1 influenza, Chima Qingwen Decoction achieved an overall efficacy rate of 93.3%, with no adverse reactions observed, supporting the feasibility of combining traditional Chinese and Western medicine in treating mild influenza	30203768
2018	Chinese Herbal Mixture	This study evaluated the efficacy and safety of a Chinese herbal mixture, finding that it effectively shortened the time to fever reduction and outperformed Western medicine in alleviating various symptoms	32185992
2018	Reduning Injection (RDN)	In pediatric influenza treatment, RDN demonstrated comparable efficacy to oseltamivir, with no severe adverse reactions, confirming the feasibility of using Chinese medicine in treating influenza in children	37474974
2021	Antiviral Granules (KBD)	KBD granules showed no significant difference from oseltamivir in terms of symptom relief and fever reduction time, but displayed certain advantages in improving specific clinical symptoms and demonstrated favorable safety	33370867
2023	Qingfei Dayuan Granules (QFDY)	In the low-dose group, QFDY demonstrated better safety and efficacy, further confirming its feasibility in the treatment of influenza	38412322
2024	Chaiyin Granules	A 2018 cost-effectiveness analysis revealed that Chaiyin granules provided no significant difference in symptom relief time compared to oseltamivir, but had a marked advantage in treatment costs, supporting the economic viability of Chinese medicine	37802879

Summarizes key findings from clinical trials on the use of TCM in influenza treatment, based on PubMed data.

A 2018 study evaluated the efficacy and safety of a TCM formulation for the treatment of seasonal influenza. The results indicated that the herbal mixture significantly shortened the time to fever reduction and was superior to Western medicines in alleviating multiple symptoms (Wu et al., 2018). This finding further supports the role of TCM in comprehensive symptom management. However, not all TCM formulations significantly enhance influenza vaccine antibody titers. A 2007 study on *Hochu-ekki-to* (HET) revealed that HET did not significantly improve antibody titers following influenza vaccination in clinical trials (Hamazaki et al., 2007). This suggests that the auxiliary effects of certain TCM preparations may be limited, warranting further investigation into their mechanisms.

In 2013, a study on *Jinhua Qinggan Granules* (JHG) in patients with wind-heat type influenza (WHAFS) demonstrated that low doses of JHG significantly reduced fever duration and alleviated symptoms, with no adverse reactions observed (Li et al., 2013). This study provided strong evidence supporting the use of TCM in treating wind-heat type influenza, particularly in alleviating its symptoms.

A 2015 study on *Banlangen Granules* (BLG) for seasonal influenza treatment showed that its therapeutic efficacy was comparable to that of oseltamivir, especially in terms of reducing fever and alleviating cough symptoms (Li Z. T. et al., 2015). This research further substantiated the competitiveness of TCM in influenza treatment. In the same year, clinical trials on *Chaihu Fufang Chima Qingwen Decoction* (CFCD) in patients with mild H1N1 influenza also yielded positive results. The study showed a 93.3% overall efficacy rate, with no adverse reactions observed during the treatment process (Yao et al., 2018). This study highlighted the feasibility of integrating TCM and Western medicine in treating mild influenza cases, further affirming the potential of TCM in influenza management.

In 2021, a multicenter clinical trial of *Anti-Viral Granules* (KBD) demonstrated that KBD was comparable to oseltamivir in terms of symptom relief and fever reduction time. However, KBD exhibited certain advantages in improving specific clinical symptoms and demonstrated good safety (Nong et al., 2021). This study provided clinical evidence supporting the use of KBD granules in influenza treatment, confirming their effectiveness in TCM-based influenza therapies. Following this, a 2023 study on *Reduning Injection* (RDN) in pediatric influenza patients showed that RDN’s efficacy was on par with oseltamivir, with no severe adverse reactions observed (Yang et al., 2023), further confirming the feasibility of TCM in treating influenza in children.

In 2024, research on *Qingfei Dayuan Granules* (QFDY) further validated their significant therapeutic effects, especially in the low-dose group, which demonstrated favorable safety and efficacy (Zhu et al., 2024). This study provided scientific evidence for the rational use of low-dose QFDY in influenza treatment, confirming its feasibility. Moreover, the pharmacoeconomic study on *Chaiyin Granules* in influenza treatment has also attracted considerable attention. A cost-effectiveness analysis conducted in 2018 revealed that *Chaiyin Granules* were comparable to oseltamivir in terms of clinical symptom relief time, yet offered a notable advantage in treatment costs (Ding et al., 2023). This finding strongly supports the economic viability of TCM in influenza treatment, particularly in large-scale applications, where it can reduce patient treatment costs.

In summary, while the efficacy and mechanisms of TCM in influenza treatment vary, most studies highlight its benefits in antiviral effects, symptom relief, and therapeutic efficacy. As clinical data grows, the potential of TCM in treating influenza becomes clearer. Future research should focus on refining its mechanisms, evaluating the safety and efficacy of TCM formulations, and expanding their clinical use in influenza management.

### 4.4 The role and prospects of traditional Chinese medicine in global influenza control

Traditional Chinese medicine (TCM) provides distinct advantages in influenza prevention and treatment through the synergistic actions of multiple components targeting diverse molecular pathways, encompassing antiviral, anti-inflammatory, and immunoregulatory effects. We compiled a summary of widely employed TCM formulations for influenza, highlighting their principal active components and documented therapeutic outcomes (Table 10). Key formulations, such as Lianhua Qingwen, inhibit H1N1 and H3N2 viral replication and suppress NF-κB-mediated inflammation, shortening febrile duration and alleviating systemic symptoms, with efficacy comparable to oseltamivir but fewer adverse effects (Xu et al., 2022; Wu et al., 2021; Du et al., 2021). Other classic prescriptions, including Yinqiao San, Shuanghuanglian, and Maxing Shigan Tang, mitigate pulmonary inflammation and restore immune balance via modulation of NLRP3 inflammasomes, TLR/NF-κB, and PI3K/AKT/mTOR pathways (Fu et al., 2018; Deng et al., 2024; Huang et al., 2023; Huang et al., 2025). Some commonly used proprietary medicines, however, are indicated primarily for wind-cold or gastrointestinal types of common cold rather than typical influenza, emphasizing the importance of syndrome-based clinical application.

**Table 10:** Key composition, active compounds, pharmacological effects, and literature support of common anti-influenza TCM formulations.

TCM formula	Key herbal ingredients (representative)	Core active compounds	Therapeutic effects	Supporting literature (PMID)
Lianhua Qingwen Capsule	Forsythia, Honeysuckle, Isatis root, Ephedra, Gypsum, Rhubarb, Agastache	Chlorogenic acid, Phillyrin, Emodin	Inhibits H1N1/H3N2 replication; suppresses NF-κB pathway; downregulates TNF-α and IL-6; reduces adhesion molecule expression, inhibiting influenza-induced bacterial adhesion	36504796; 34306428; 33872750
Yinqiao San	Honeysuckle, Forsythia, Mint, Schizonepeta, Arctium, Fermented soybean	Luteolin, Chlorogenic acid, Quercetin	Promotes mitophagy, reduces ROS, inhibits NLRP3 inflammasome; downregulates TLR7/NF-κB signaling	38518643; 30151032
Shuanghuanglian Oral Liquid	Honeysuckle, Scutellaria, Forsythia	Baicalin, Chlorogenic acid, Forsythiaside	Suppresses TNF-α, IL-1β, and IL-6 release via TNF signaling; inhibits viral neuraminidase activity	33276058; 21351522; 40154897
Banlangen Granules	Isatis root	Indirubin, Indigo, Polysaccharides	Inhibits H1N1-induced pro-inflammatory cytokines (IL-6, TNF-α, IL-8, MCP-1, IP-10, IFN-α); attenuates NF-κB activation and TLR3 signaling; clinically comparable to oseltamivir in seasonal influenza, with superior antipyretic and antitussive effects	26320688; 34543684; 25873046
Maxing Shigan Decoction	Ephedra, Apricot seed, Gypsum, Licorice	Ephedrine, Amygdalin, Glycyrrhizic acid	Modulates PI3K/AKT/mTOR pathway to suppress hyperinflammation; reduces viral load, oxidative stress, and ferroptosis	40659142; 37879795
Qingkailing Injection	Cholic acid, Pearl, Hyodeoxycholic acid, Buffalo horn, Scutellaria, Honeysuckle, Gardenia	Baicalin, Chlorogenic acid, Bile acids	Downregulates PI3K/AKT and SRC/STAT3 pathways to alleviate lung inflammation; inhibits viral neuraminidase	38537336; 21351522
Jinhua Qinggan Granules	Honeysuckle, Fritillaria, Scutellaria, Artemisia, Ephedra, Apricot seed, Gypsum, Licorice	Chlorogenic acid, Baicalin, Ephedrine	Inhibits TLR4/MyD88/NF-κB pathway, reducing pulmonary inflammation; shortens fever duration and alleviates wind-heat flu symptoms with good safety	24517059; 36183949
Chaiyin Granules	Bupleurum, Honeysuckle, Scutellaria, Forsythia, Platycodon, Licorice	Saikosaponins, Baicalin, Chlorogenic acid	Comparable efficacy to oseltamivir but significantly lower treatment cost, offering economic advantage	37802879
Kangbingdu Granules	Isatis root, Isatis leaf, Forsythia, Notopterygium, *Polygonum bistorta*	Indirubin, Phillyrin, Catechin	Symptom relief and fever resolution comparable to oseltamivir; shows advantages in certain clinical outcomes with favorable safety	33370867; 37567423
Qingfei Dayuan Granules	Bupleurum, Scutellaria, Magnolia bark, Amomum, Areca, Artemisia, Anemarrhena, Red peony, Licorice	Saikosaponins, Baicalin, Artemisinin derivatives	Inhibits TNF-α and IL-1β production; suppresses TLR4-TRIF/MyD88-NF-κB-NLRP3 pathway; low-dose regimen shows optimal safety and efficacy for influenza treatment	41311834; 38412322
Reduning Injection	Artemisia, Honeysuckle, Gardenia	Artesunate, Chlorogenic acid, Geniposide	Suppresses excessive inflammatory cytokine production; as effective as oseltamivir in pediatric influenza with no serious adverse events	36164877; 37474974
Bu-Zhong-Yi-Qi-Tang (Hochu-ekki-to, HET)	Astragalus, Ginseng, Atractylodes, Licorice, Angelica, Tangerine peel, Cimicifuga, Bupleurum, Ginger, Jujube	Astragaloside IV, Ginsenosides, Glycyrrhizic acid	Enhances type I interferon and antimicrobial peptide responses; inhibits influenza virus replication; shows limited adjuvant effect on vaccine-induced antibody responses	23796966; 16644196
Qiwu Qingwen Decoction (Chima Qingwen)	Honeysuckle, Forsythia, Scutellaria, Isatis root, Mint, Platycodon, Licorice (inferred from “Qingwen” class formulas)	Chlorogenic acid, Baicalin, Forsythiaside	Achieves 93.3% overall efficacy in mild H1N1 influenza; safe and supports integrative Chinese-Western medicine treatment	30203768

This table summarizes essential information for clinically used TCM anti-influenza formulations, including representative herbal components, major bioactive compounds, principal pharmacological effects (e.g., antiviral, anti-inflammatory, immunomodulatory), and supporting references (PMID).

The use of botanical remedies for respiratory infections extends beyond TCM. Ayurveda employs *Ocimum sanctum* (Tulsi) and *Tinospora cordifolia* (Giloy) for seasonal fevers, with *Withania somnifera* (WS) and *Tinospora cordifolia* (TC) exhibiting potent immunomodulatory effects, utilized by the Indian AYUSH authority during the COVID-19 pandemic (Prakash and Gupta, 2005; Devpura et al., 2021; Rizvi et al., 2023). In Korea, Kampo-like formulations such as Kakkon-to and Mahuang-Fuzi-Xixin Tang are used for influenza-like illnesses, integrated into the national health insurance system (Jeong et al., 2014). In Japan, TCM-derived Kampo prescriptions are regulated and studied for antiviral and immunomodulatory mechanisms (Takanashi et al., 2017; Nagai et al., 1996). In Southeast Asia, herbs such as turmeric, *Andrographis paniculata*, and lemongrass are commonly used to relieve influenza symptoms, with constituents like andrographolide demonstrating inhibition of viral replication (Siridechakorn et al., 2023). Despite theoretical differences, these traditional systems converge on prevention, individualized therapy, and natural-product-based interventions, providing complementary strategies for influenza control.

Future global integration of TCM requires rigorous scientific validation, international collaboration, and standardized clinical and quality guidelines. Multi-omics and systems pharmacology approaches can clarify the “multi-component, multi-target” mechanisms of TCM formulations, enabling a shift from empirical use to precision therapeutics. Strengthening exchanges with Ayurveda and Korean/Japanese Kampo medicine will support internationally recognized clinical evidence, quality standards, and syndrome-based guidelines, positioning TCM as a globally relevant strategy in public health influenza prevention and treatment.

### 4.5 Clinical translational potential of key research hotspots in TCM for influenza

Influenza-focused TCM research is shifting from empirical efficacy studies toward mechanistic investigation. Recent hotspots include immune regulation, virus-related inflammatory pathways, gut microbiota, and network pharmacology, reflecting closer integration with modern biomedical approaches. Most TCM formulations act by modulating host immunity rather than directly inhibiting viral replication, particularly via suppression of NF-κB and NLRP3 inflammasomes, reducing excessive inflammation and lung injury (Xu et al., 2022; Zhou et al., 2023).

The gut–lung axis has emerged as a key target, with some TCMs restoring immune balance through microbiota modulation, supporting individualized therapy (Sencio et al., 2021). Systems biology tools, such as network pharmacology and molecular docking, facilitate mapping multi-component, multi-target networks, guiding formulation optimization and rational combination with Western antivirals. Future integration of TCM into modern influenza care requires biomarker-based syndrome differentiation, high-quality clinical trials, and validation of precise therapeutic effects, enabling a transition from empirical use to evidence-based, integrative medicine.

### 4.6 International collaboration: challenges and opportunities

International collaboration in TCM influenza research (10.23%) lags behind Western antivirals (20.13%), due to language barriers, differences in theoretical frameworks, lack of formulation standardization, and limited cross-border mechanisms. Many high-quality studies are published in Chinese journals with minimal English abstracts, limiting global access. TCM emphasizes holistic syndrome-based therapy, whereas Western medicine prioritizes target-mechanism-validation, creating differences in study design and evidence standards.

Western antivirals follow a “single-target, high-potency” model (e.g., oseltamivir inhibits neuraminidase), while TCM uses a “multi-component, multi-target” approach, modulating host immunity, microenvironment, and virus-bacteria interactions. Although these differences complicate collaboration, they also create opportunities. Leveraging modern pharmacology and AI, TCM can identify antiviral leads, design synergistic TCM-Western therapies, and support multicenter international trials. Building global networks and multilingual knowledge platforms will help TCM align with international research standards, enhancing its role in respiratory disease prevention and as a source of novel therapeutics.

## 5 Limitations

This study has several methodological limitations that should be considered when interpreting the results. First, to ensure standardization and cross-study comparability of citation data, the analysis focused exclusively on English-language publications. This approach enhances international readability and methodological consistency but inevitably excludes some studies published in regional languages such as Chinese, Japanese, or Korean. Although these local studies hold value within specific contexts, their visibility in the global citation network is relatively limited. Integrating multilingual databases such as CNKI or CiNii in future studies could provide a more comprehensive global perspective on TCM research.

Second, WOSCC and Scopus differ in coverage, update frequency, and document types: WOSCC offers a longer historical record and more stable citation structures, whereas Scopus has broader coverage and faster updates. Cross-deduplication and field standardization were applied to balance these advantages. Nevertheless, differences in indexing logic may still slightly affect the identification of institutional affiliations or collaboration networks. Additionally, the dataset only includes publications up to November 2025, and standardization of author or institution names is limited by current bibliometric tools. However, given the large sample size and extensive temporal span, these factors are unlikely to substantially alter the observed trends.

It is important to note that bibliometric analysis provides a macro-level, retrospective overview of research evolution rather than a substitute for clinical or mechanistic studies. Within this context, the present study offers an objective and quantitative reference for understanding the differentiated development of TCM and Western antiviral research in the global influenza landscape.

## 6 Conclusion

This study utilized bibliometric and content analysis methods to evaluate the progress of research on traditional Chinese medicine (TCM) in the treatment of influenza from 2000 to 2025. The results indicate a steady growth in this field, with an average annual growth rate of 16.94%. Notably, following the COVID-19 pandemic (after 2019), research on TCM for influenza treatment saw an explosive increase, reflecting the global attention on the role of TCM interventions in respiratory viral infections. China has dominated this field, contributing nearly two-thirds of the literature, with institutions such as Beijing University of Chinese Medicine forming core research clusters, thereby further solidifying China’s scientific position. However, the proportion of international collaboration remains low (only 10.23%), highlighting the need for increased cross-national cooperation, particularly in multi-center clinical trials and mechanistic studies. The research focus has gradually shifted from empirical formula screening to modern scientific approaches, such as network pharmacology, molecular docking, and gut microbiome regulation, reflecting the trend of integrating “traditional experience—modern mechanisms—clinical translation.” Current research hotspots are centered on antiviral activity, immune regulation, oxidative stress, and multi-target synergy. High-impact studies, such as those on Lianhua Qingwen, have advanced the development of integrative medicine, enhancing the influence of TCM in global public health. While clinical trials have demonstrated that several TCM formulations offer comparable or superior efficacy and cost-effectiveness to oseltamivir in fever reduction and cough relief, there remains a lack of large-scale, high-quality, evidence-based studies.

## Data Availability

The original contributions presented in the study are included in the article/Supplementary material, further inquiries can be directed to the corresponding authors.
