# Supplementary material for: Traditional Chinese medicine in influenza treatment: a bibliometric analysis integrating multiple databases
Source: Front Microbiol. 2026 Mar 12;17:1761339. doi: 10.3389/fmicb.2026.1761339 (PMC13018163; doi:10.3389/fmicb.2026.1761339)
Supplement: Supplementary file 1 [file Table_1.docx]

Supplementary Material

# Supplementary Figures and Tables

For more information on Supplementary Material and for details on the different file types accepted, please see [here](https://www.frontiersin.org/guidelines/author-guidelines#supplementary-material).

## Supplementary Figures


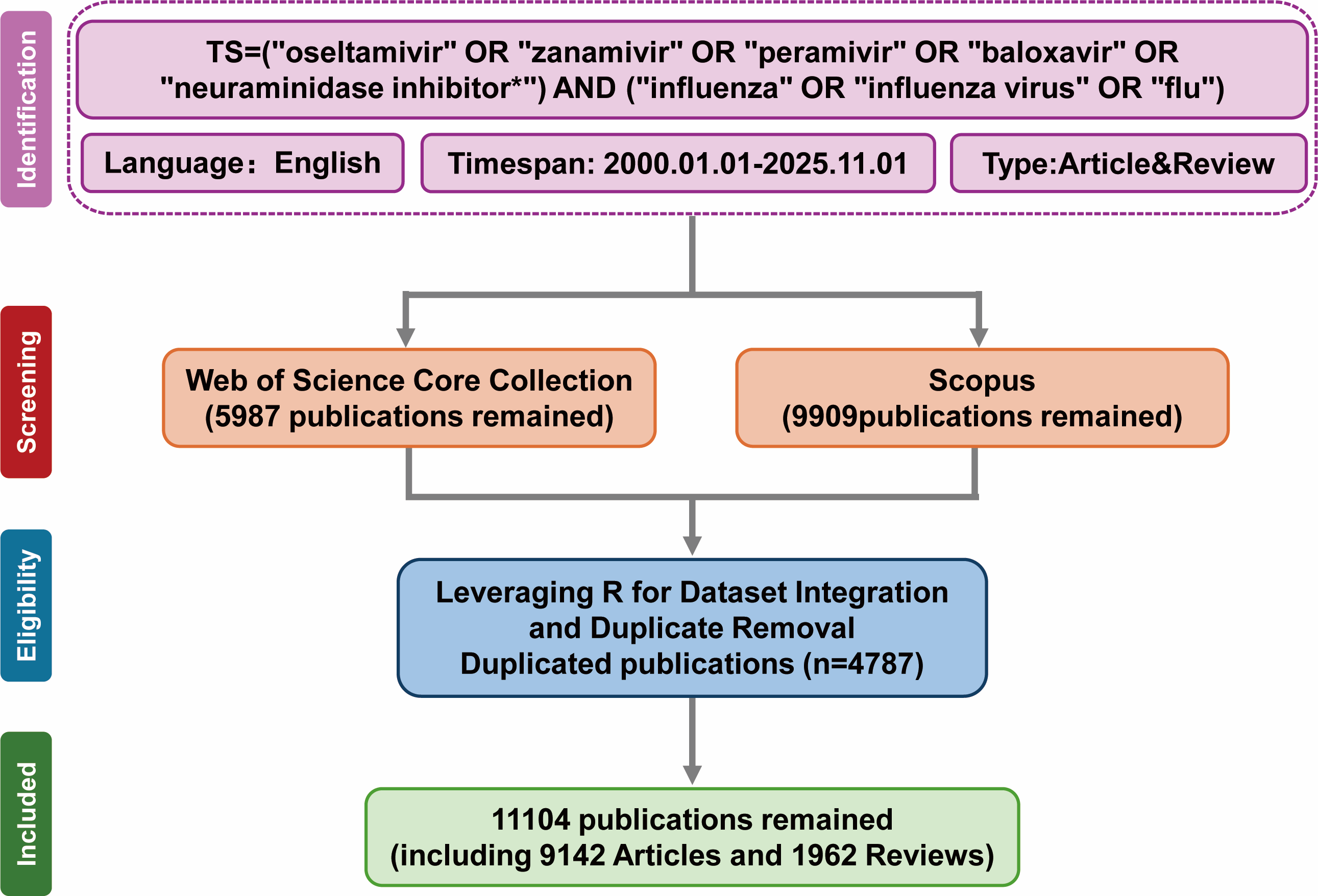


**Supplementary Figure 1. Literature Retrieval and Data Cleaning Workflow for Western Antiviral Drugs in Influenza Research**

Illustrates the process of retrieving English-language literature (2000–2025) on neuraminidase inhibitors such as oseltamivir for influenza treatment. Initial searches yielded 5,987 records from Web of Science Core Collection (WOSCC) and 9,909 records from Scopus. After merging and removing 4,787 duplicates, relevance screening resulted in 11,104 articles included for comparative analysis with TCM studies.

## Supplementary Tables

**Supplementary Table 1. Retrieval Strategy for TCM Studies on Influenza (WOSCC, Scopus, PubMed)**

| **Database** | **Search Strategy** | **Search Date** | **Filtering Conditions** |
| --- | --- | --- | --- |
| **WOSCC** | TS1=("Traditional Chinese Medicine" OR "TCM" OR "Chinese herbal medicine" OR "herbal formula*" OR "herbal remedy*")  TS2=("Influenza" OR "Flu" OR "respiratory virus*" OR "respiratory infection*")  TS=TS1 AND TS2 | November 1, 2025 | English-language publications from January 1, 2000, to November 1, 2025; includes Article and Review Article types |
| **Scopus** | (TITLE-ABS-KEY (("Traditional Chinese Medicine" OR "TCM" OR "Chinese herbal medicine" OR "herbal formula*" OR "herbal remedy*")) AND TITLE-ABS-KEY (("Influenza" OR "Flu" OR "respiratory virus*" OR "respiratory infection*"))) AND PUBYEAR > 1999 AND PUBYEAR < 2026 AND (LIMIT-TO (DOCTYPE, "ar") OR LIMIT-TO (DOCTYPE, "re")) AND (LIMIT-TO (LANGUAGE, "English")) | November 1, 2025 | English-language publications from January 1, 2000, to November 1, 2025; includes Article and Review Article types |
| **PubMed** | (("Traditional Chinese Medicine"[Title/Abstract] OR "TCM"[Title/Abstract] OR "Chinese herbal medicine"[Title/Abstract] OR "herbal formula*"[Title/Abstract] OR "herbal remedy*"[Title/Abstract]) AND ("Influenza"[Title/Abstract] OR "Flu"[Title/Abstract] OR "respiratory virus*"[Title/Abstract] OR "respiratory infection*"[Title/Abstract])) AND ("2000/01/01"[Date - Publication] : "2025/11/01"[Date - Publication]) | November 1, 2025 | Clinical Trial studies published between January 1, 2000, and November 1, 2025 |

Details the specific search strategies employed across WOSCC, Scopus, and PubMed, including inclusion criteria, keyword combinations, and screening parameters, to ensure comprehensive and rigorous acquisition of literature on TCM interventions for influenza.

**Supplementary Table 2. Retrieval Strategy for Western Antiviral Drugs in Influenza Research (WOSCC and Scopus)**

| **Database** | **Search Strategy** | **Filtering Conditions** |
| --- | --- | --- |
| **WOSCC** | TS1= ("oseltamivir" OR "zanamivir" OR "peramivir" OR "baloxavir" OR "neuraminidase inhibitor*")  TS2=("influenza" OR "influenza virus" OR "flu")  TS=TS1 AND TS2 | English-language publications from January 1, 2000, to November 1, 2025; includes Article and Review Article types |
| **Scopus** | (TITLE-ABS-KEY (("oseltamivir" OR "zanamivir" OR "peramivir" OR "baloxavir" OR "neuraminidase inhibitor*")) AND TITLE-ABS-KEY (("influenza" OR "influenza virus" OR "flu"))) AND PUBYEAR > 1999 AND PUBYEAR < 2026 AND (LIMIT-TO (DOCTYPE, "ar") OR LIMIT-TO (DOCTYPE, "re")) AND (LIMIT-TO (LANGUAGE, "English")) | English-language publications from January 1, 2000, to November 1, 2025; includes Article and Review Article types |

Outlines the search strategies used in WOSCC and Scopus, including inclusion criteria and keywords (e.g., oseltamivir, zanamivir, baloxavir, neuraminidase inhibitor, influenza), to systematically capture relevant publications on Western antiviral drugs.

**Supplementary Table 3. Overall Comparison of TCM and Western Antiviral Research on Influenza (Based on Combined WOSCC and Scopus Data)**

| **Description** | **TCM research in influenza** | **Western antiviral drug research in influenza** |
| --- | --- | --- |
| Timespan | 2000:2025 | 2000:2025 |
| Sources (Journals, Books, etc) | 407 | 2381 |
| Documents | 959 | 11104 |
| Annual Growth Rate % | 16.94 | 5.03 |
| Document Average Age | 6.16 | 10.4 |
| Average citations per doc | 27.4 | 38.47 |
| Keywords Plus (ID) | 7959 | 29560 |
| Author's Keywords (DE) | 2567 | 12642 |
| Authors | 2915 | 52054 |
| Authors of single-authored docs | 27 | 660 |
| Single-authored docs | 27 | 939 |
| Co-Authors per Doc | 7.11 | 6.88 |
| International co-authorships % | 10.23 | 20.13 |
| article | 747 | 9142 |
| review | 212 | 1962 |

Summarizes multiple indicators for the two research categories from 2000–2025, including average annual publication growth rate (TCM: 16.94%; Western drugs: 5.03%) and international collaboration rate (TCM: 10.23%; Western drugs: 20.13%), reflecting differences in research pace, scientific activity, and global collaboration.

**Supplementary Table 4. Annual Mean Citations per Article (Mean TC per Article) for TCM and Western Antiviral Research**

| **Year** | **TCM–influenza (Mean TC/article)** | **Western antivirals–influenza (Mean TC/article)** |
| --- | --- | --- |
| 2000 | 45.00 | 63.62 |
| 2001 | 18.00 | 70.14 |
| 2002 | 51.00 | 57.74 |
| 2003 | 147.40 | 63.80 |
| 2004 | 86.83 | 100.46 |
| 2005 | 66.08 | 92.85 |
| 2006 | 54.29 | 70.00 |
| 2007 | 27.00 | 78.93 |
| 2008 | 48.40 | 58.81 |
| 2009 | 152.12 | 83.94 |
| 2010 | 48.55 | 45.55 |
| 2011 | 50.87 | 32.28 |
| 2012 | 23.91 | 38.59 |
| 2013 | 36.19 | 46.95 |
| 2014 | 32.81 | 35.17 |
| 2015 | 43.90 | 33.21 |
| 2016 | 37.87 | 30.09 |
| 2017 | 51.79 | 29.95 |
| 2018 | 40.72 | 30.10 |
| 2019 | 21.46 | 25.55 |
| 2020 | 45.25 | 38.63 |
| 2021 | 26.48 | 23.44 |
| 2022 | 12.38 | 13.32 |
| 2023 | 9.25 | 9.25 |
| 2024 | 4.90 | 4.62 |
| 2025 | 0.70 | 1.34 |

Displays the yearly average citations per article for both research categories since 2000, evaluating the temporal evolution of their academic impact.

**Supplementary Table 5. Top 10 Countries by Publication Volume and International Collaboration in Western Antiviral Research on Influenza**

| **Country** | **Articles** | **Articles %** | **SCP** | **MCP** | **MCP %** |
| --- | --- | --- | --- | --- | --- |
| USA | 2423 | 21.8 | 1938 | 485 | 20 |
| CHINA | 1786 | 16.1 | 1511 | 275 | 15.4 |
| JAPAN | 894 | 8.1 | 740 | 154 | 17.2 |
| UNITED KINGDOM | 483 | 4.3 | 358 | 125 | 25.9 |
| INDIA | 412 | 3.7 | 365 | 47 | 11.4 |
| CANADA | 366 | 3.3 | 271 | 95 | 26 |
| AUSTRALIA | 335 | 3 | 232 | 103 | 30.7 |
| KOREA | 326 | 2.9 | 287 | 39 | 12 |
| GERMANY | 235 | 2.1 | 174 | 61 | 26 |
| ITALY | 220 | 2 | 169 | 51 | 23.2 |

Lists the top ten countries in Western antiviral influenza research by publication count and their respective international collaboration rates, reflecting participation in the global research network.

**Supplementary Table 6. High-Output Authors and Research Impact Metrics in Western Antiviral Influenza Research**

| **Author** | **h_index** | **g_index** | **m_index** | **TC** | **NP** | **PY_start** |
| --- | --- | --- | --- | --- | --- | --- |
| GUBAREVA LARISA V. | 39 | 62 | 1.95 | 5200 | 62 | 2006 |
| GOVORKOVA ELENA A. | 36 | 61 | 1.8 | 3831 | 72 | 2006 |
| WEBSTER ROBERT G. | 33 | 47 | 1.65 | 4650 | 47 | 2006 |
| BOIVIN GUY | 32 | 55 | 1.6 | 3132 | 67 | 2006 |
| HURT AERON C. | 32 | 66 | 1.684 | 4492 | 69 | 2007 |
| UYEKI TIMOTHY M | 32 | 48 | 1.333 | 12802 | 48 | 2002 |
| HAYDEN FREDERICK G. | 31 | 39 | 1.55 | 6620 | 39 | 2006 |
| FRY ALICIA M. | 28 | 50 | 1.647 | 4226 | 50 | 2009 |
| HAYDEN FG | 28 | 32 | 1.077 | 8278 | 32 | 2000 |
| LACKENBY ANGIE | 25 | 36 | 1.316 | 3160 | 36 | 2007 |

Presents the top ten most prolific authors in the field, along with metrics of scholarly influence, including h-index, g-index, m-index, and total citations.

**Supplementary Table 7. High-Impact Journals in Western Antiviral Influenza Research and Their Metrics**

| **Source** | **h_index** | **g_index** | **m_index** | **TC** | **NP** | **PY_start** |
| --- | --- | --- | --- | --- | --- | --- |
| CLINICAL INFECTIOUS DISEASES | 66 | 136 | 2.538 | 18748 | 154 | 2000 |
| ANTIVIRAL RESEARCH | 63 | 108 | 2.423 | 14146 | 275 | 2000 |
| ANTIMICROBIAL AGENTS AND CHEMOTHERAPY | 56 | 95 | 2.154 | 9733 | 146 | 2000 |
| JOURNAL OF INFECTIOUS DISEASES | 55 | 91 | 2.115 | 8841 | 137 | 2000 |
| PLOS ONE | 52 | 77 | 2.737 | 9635 | 277 | 2007 |
| EMERGING INFECTIOUS DISEASES | 49 | 89 | 2.13 | 8632 | 144 | 2003 |
| JOURNAL OF VIROLOGY | 48 | 83 | 1.846 | 7284 | 109 | 2000 |
| JOURNAL OF MEDICINAL CHEMISTRY | 41 | 65 | 1.577 | 4296 | 73 | 2000 |
| NEW ENGLAND JOURNAL OF MEDICINE | 37 | 55 | 1.423 | 20258 | 55 | 2000 |
| INFLUENZA AND OTHER RESPIRATORY VIRUSES | 34 | 58 | 1.789 | 4360 | 198 | 2007 |

Identifies the top ten journals publishing relevant studies, providing associated influence metrics including h-index, g-index, m-index, and total citations.
